# Supplementary material for: Structural perspectives on adenosine to inosine RNA editing by ADARs
Source: Mol Ther Nucleic Acids. 2024 Jul 19;35(3):102284. doi: 10.1016/j.omtn.2024.102284 (PMC11334849; doi:10.1016/j.omtn.2024.102284)
Supplement: Document S1. Figures S1–S3 [file mmc1.pdf]

OMTN, Volume *35*

Supplemental information

Structural perspectives on adenosine to inosine RNA editing by ADARs

Andrew J. Fisher and Peter A. Beal

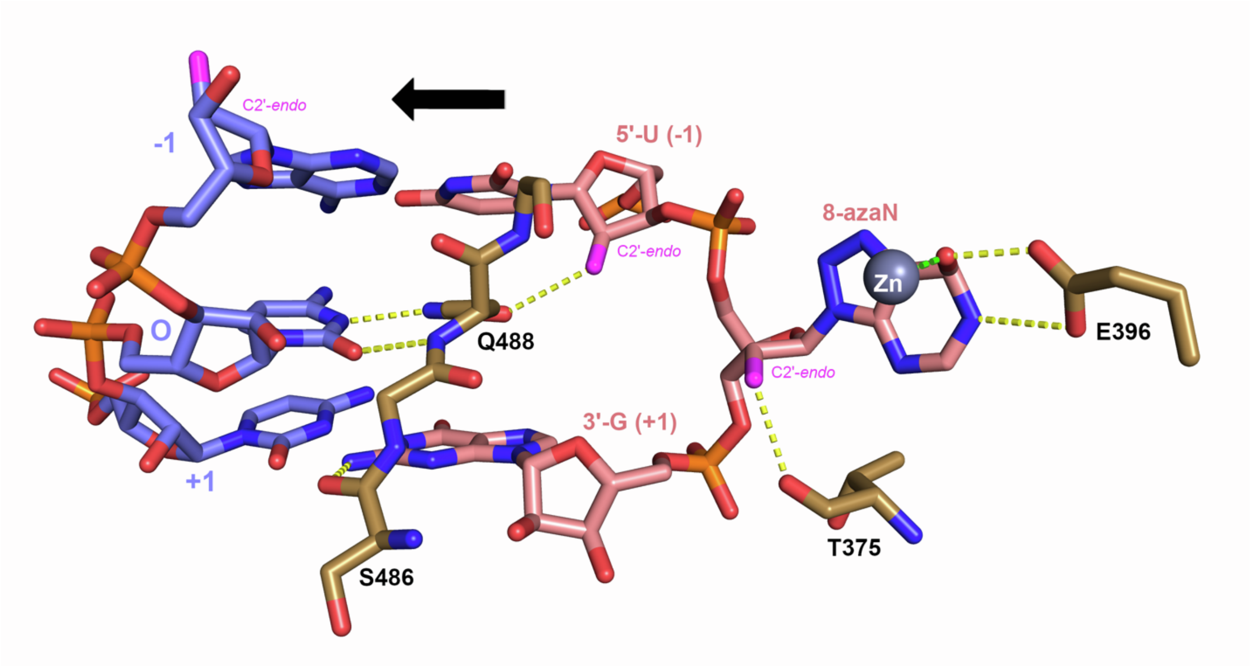

Figure S1. *Detailed RNA-Protein contacts near the flipped-out base. Edited RNA strand is shown in salmon-colored carbon atoms, guide strand RNA in blue-colored carbon. RNA with 2'C-endo sugar puckers are highlighted with magenta-colored 2'-O atom and labeled. Guild strand RNA numbering shown in blue with the -1 position base-pairing to the 5'-U of the edited strand. Bold black arrow shows "sliding" of 5'-end base-pair relative to ideal A-form conformation. Yellow dashed lines show hydrogen bonding, green dash, zinc ligation.*

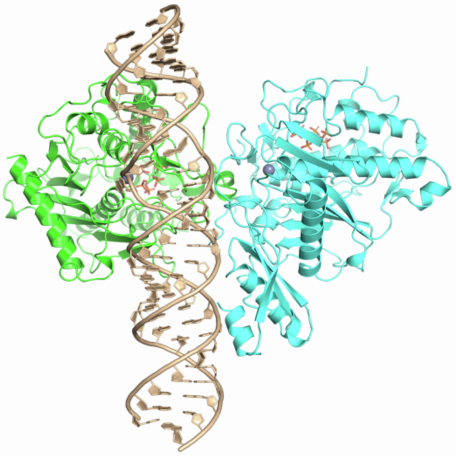

X-ray Structure of ADAR2-R2D w/ dsRNA, PDB: 6vff

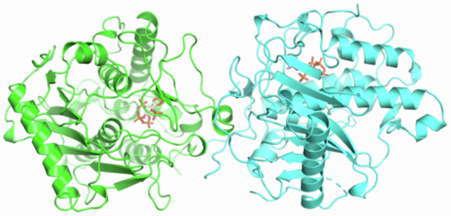

X-ray Structure of ADAR2d w/o dsRNA, PDB: 1zy7

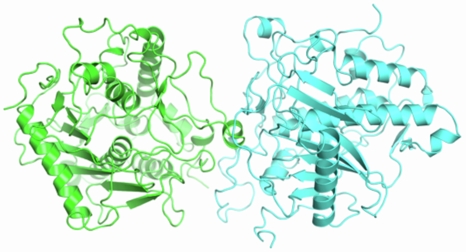

AlphaFold2 Predicted Dimer of ADAR2d

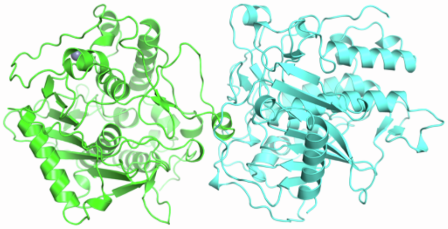

AlphaFold2 Predicted Dimer of ADAR1d

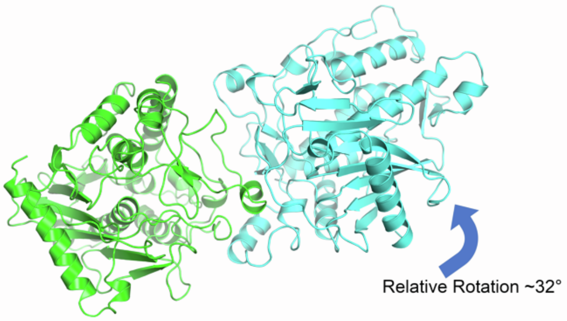

AlphaFold2 Predicted Dimer of ADAR3d

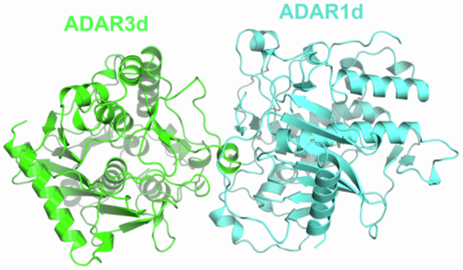

AlphaFold2 Predicted
ADAR3d-ADAR1d Heterodimer

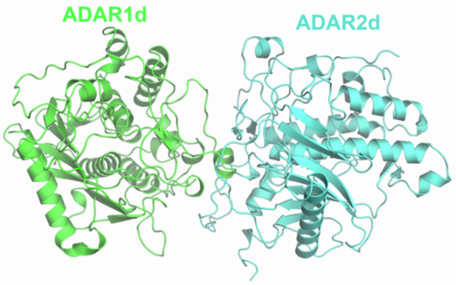

AlphaFold2 Predicted
ADAR1d-ADAR2d Heterodimer

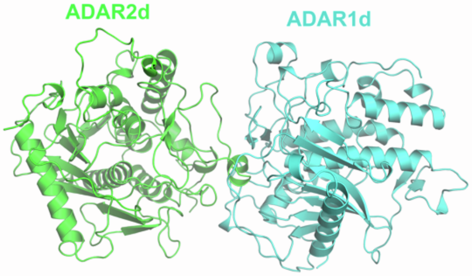

AlphaFold2 Predicted
ADAR2d-ADAR1d Heterodimer

Figure S2. *Dimeric arrangement of ADARs. Shown are different ADAR structures determined experientially and modeled by AlphaFold2 showing the dimerization helix mediates similar asymmetric homodimers from different ADARs. Green-colored monomers reside in the catalytic position whose active site would bind the flipped-out adenosine, and the cyan-colored monomers represent the auxiliary position, whose active site binds the catalytic monomer's dimerization helix. AlphaFold2 also predicts an ADAR3d-ADAR2d heterodimer, but this not shown.*

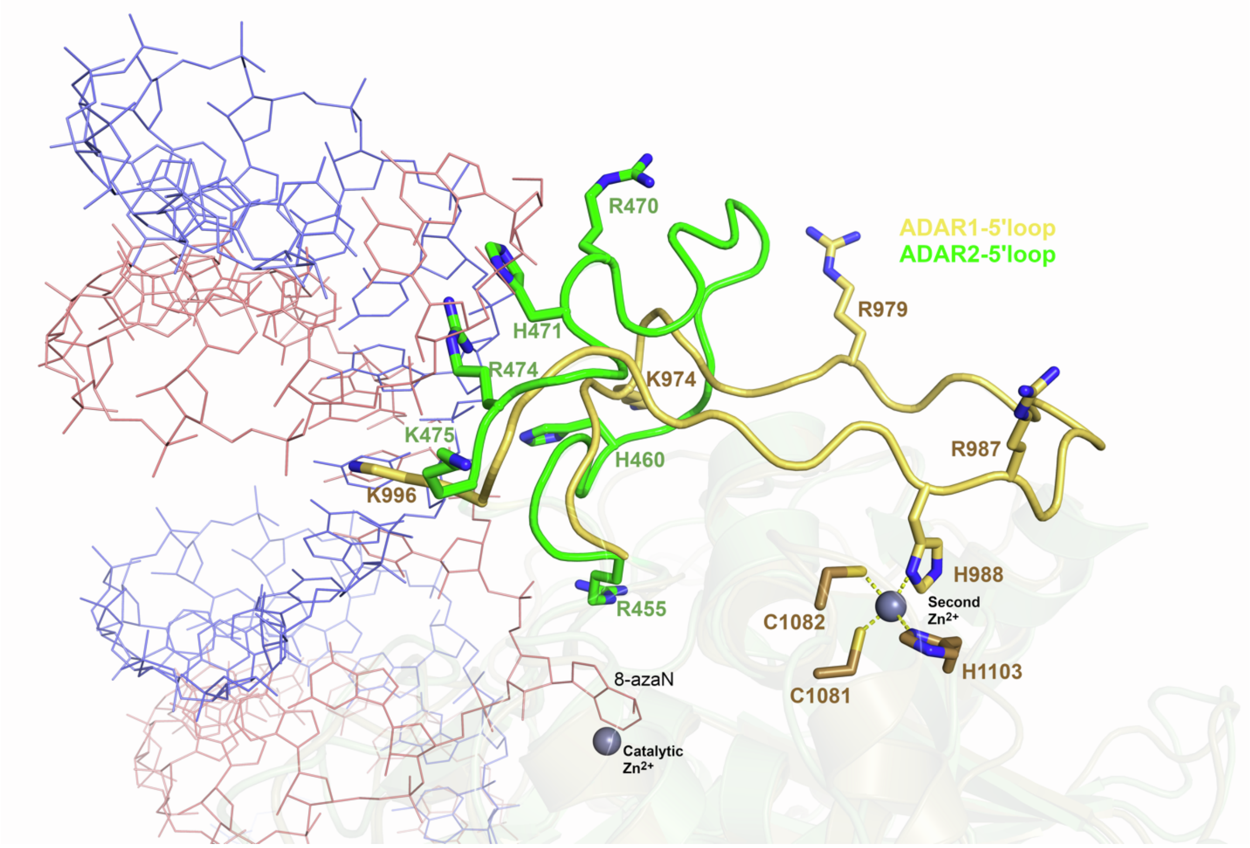

Figure S3. *Structures of the 5'-RNA binding loop as seen in the crystal structure of ADAR2 (Green, PDBID: 6vff), and the AlphaFold2 predicted structure of human ADAR1 (yellow). Only the loops are highlighted with the rest of the protein structures shown in transparent cartoon (green and brown for ADAR2 and ADAR1, respectively) and the RNA in the ADAR2-RD structure displayed in thin lines. Conserved residues in the 5'-RNA binding loop for ADAR2 that interact with RNA are shown as stick sidechains. Positively-charged conserved residues in ADAR1 are also shown with stick sidechains.*
