# Supplementary material for: Structural perspectives on adenosine to inosine RNA editing by ADARs
Source: Mol Ther Nucleic Acids. 2024 Jul 19;35(3):102284. doi: 10.1016/j.omtn.2024.102284 (PMC11334849; doi:10.1016/j.omtn.2024.102284)
Supplement: Document S2. Article plus supplemental information [file mmc2.pdf]

Molecular Therapy
Nucleic Acids
Review

Structural perspectives on adenosine to inosine RNA editing by ADARs

Andrew J. Fisher[1,2] and Peter A. Beal[1]

[1]Department of Chemistry, University of California, Davis, One Shields Ave, Davis, CA 95616, USA; [2]Department of Molecular and Cellular Biology, University of California, Davis, One Shields Ave, Davis, CA 95616, USA

Adenosine deaminases acting on RNA (ADARs) are enzymes that catalyze the hydrolytic deamination of adenosine to inosine. The editing feature of ADARs has garnered much attention as a therapeutic tool to repurpose ADARs to correct disease-causing mutations at the mRNA level in a technique called site-directed RNA editing (SDRE). Administering a short guide RNA oligonucleotide that hybridizes to a mutant sequence forms the requisite dsRNA substrate, directing ADARs to edit the desired adenosine. However, much is still unknown about ADARs' selectivity and sequence-specific effects on editing. Atomic-resolution structures can help provide additional insight to ADARs' selectivity and lead to novel guide RNA designs. Indeed, recent structures of ADAR domains have expanded our understanding on RNA binding and the base-flipping catalytic mechanism. These efforts have enabled the rational design of improved ADAR guide strands and advanced the therapeutic potential of the SDRE approach. While no full-length structure of any ADAR is known, this review presents an exposition of the structural basis for function of the different ADAR domains, focusing on human ADAR2. Key insights are extrapolated to human ADAR1, which is of substantial interest because of its widespread expression in most human tissues.

INTRODUCTION

RNA undergoes many different forms of modification and editing events after transcription.[1–4] Currently, over 170 different types of RNA modifications have been reported in all domains of life.[5] The modified nucleosides can alter RNA secondary structure by changing hydrogen-bonding patterns, affecting base-stacking potential, or favoring a specific nucleotide conformation. Modifications can also alter recognition determinants impacting interactions with cellular components. Some changes can also recode mRNA by altering the genetic interpretation of the base, which is referred to as "RNA editing." RNA modifications and editing events play critical roles in several important cellular processes and regulation,[6,7] and aberrant regulation of RNA modification has been linked to different phenotypes and diseases in higher eukaryotes.[8–10]

One common type of RNA modification is methylation of the nucleobases and/or sugars.[11,12] This modification is carried out by methyltransferases, or "writers," usually utilizing *S*-adenosyl-L-methionine as the methyl donor. Cellular proteins can often recognize this RNA modification by "reader" proteins, which play important roles for proper RNA regulation and function by recruiting other cellular factors. RNA methylation can also be reversible through demethylase "erasers" enzymes.[13]

RNA editing generally refers to alterations in the RNA that irreversibly change the nucleotide sequence by mechanisms other than RNA splicing. The most frequent type of RNA editing arises from the hydrolytic deamination of either cytidine (C) or adenosine (A), producing uridine (U) or inosine (I) nucleotides, respectively. A-to-I editing in RNA is more widespread than C-to-U and occurs in all domains of life[14,15] and is the focus of this review. The A-to-I edits can alter the secondary structure of RNAs,[16] which has been implicated in averting "self" dsRNA auto-immunopathology events.[17–19] However, this nucleobase deamination chemistry can have enormous consequences in RNA function through discerned sequence changes. Edits in RNA have been shown to regulate and alter pre-mRNA splicing events.[20,21] Moreover, edits in the coding region of mRNA can alter codons, translating a protein with an amino acid substitution, thus expanding the proteome beyond the DNA coding sequence.[22] One of the first discovered examples of A-to-I RNA editing in the coding region of a protein resulting in an amino acid substitution is the glutamine to arginine change (Q/R site) in the glutamate receptor (GluA2).[23] This radical substitution controls the calcium permeability of the AMPA (α-amino-3-hydroxy-5-methyl-4-isoxazole propionate) receptor, and editing of its mRNA is essential for life.[24] Since then, there has been an expanding list of ADAR-mediated recoding events in mRNA.[25,26]

The enzyme that catalyzes this A-to-I RNA edit is called adenosine deaminase acting on RNA (ADAR), which recognizes and edits specific adenosines in regions of double-stranded RNA.[27] Inosine, which is structurally similar to guanosine but lacks guanosine's exocyclic amine, can base pair with cytidine and is typically recognized by

https://doi.org/10.1016/j.omtn.2024.102284.

Correspondence: Andrew J. Fisher, Department of Chemistry, University of California, Davis, One Shields Ave, Davis, CA 95616, USA.
E-mail: ajfisher@ucdavis.edu
Correspondence: Peter A. Beal, Department of Chemistry, University of California, Davis, One Shields Ave, Davis, CA 95616, USA.
E-mail: pabeal@ucdavis.edu

© 2024 The Author(s). Published by Elsevier Inc. on behalf of The American Society of Gene and Cell Therapy.
This is an open access article under the CC BY license (http://creativecommons.org/licenses/by/4.0/).

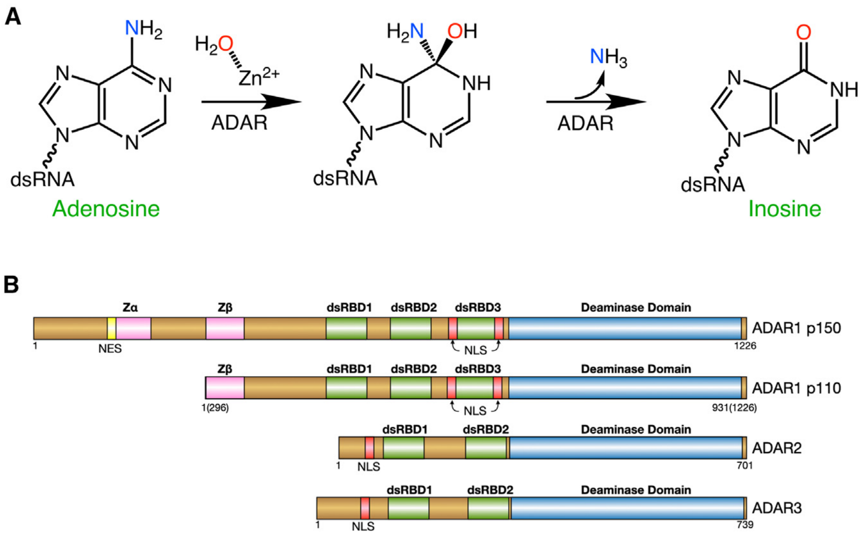

Figure 1. ADAR reaction and architecture

(A) Catalytic mechanism of ADARs. The hydrolytic deamination proceeds through a two-step mechanism with a tetrahedral intermediate. (B) Domain architecture of human ADARs. ADAR1 and ADAR 2 are catalytically active, while ADAR3 is expressed mainly in the brain but is inactive. All ADARs contain a conserved catalytic deaminase domain (blue) and dsRNA binding domains (green), and ADAR1 contains a Z-alpha domain (pink).

the cellular machinery as guanosine producing an A-to-G exchange at the RNA level. ADARs evoke a base-flipping mechanism, where the targeted adenosine is flipped out of the dsRNA helix into the ADAR active site.[28] ADARs contain an active site zinc ion, which is ligated by two cysteines and one histidine from the enzyme, with the fourth zinc ligand being a water molecule, which attacks carbon-6 of the adenine base, releasing the 6-amino group producing inosine (Figure 1A). Higher eukaryotes possess two catalytically active ADARs, ADAR1 and ADAR2, which contain similar modular domain arrangements. Both ADARs are essential for life since knock-outs in mice are lethal.[29] However, ADAR knockouts can be rescued by distinct downstream manipulations.[30,31] In this review, we will focus on the structural basis for RNA editing by human ADARs, concentrating on human ADAR2 where crystal structures are available and projecting some key observations to ADAR1, for which there are no published structures of the deaminase domain at this time but where AlphaFold models help support some conclusions.

ADAR DOMAIN ARCHITECTURE

ADARs, found in all metazoans, have a modular architecture with highly conserved domains.[32,33] All ADARs harbor a deamination domain comprising roughly the C-terminal 400 residues, but the N-terminal domains differ based on ADAR type. Vertebrates typically have three types, or paralogs, of ADARs (Figure 1B). ADAR1 and ADAR2 are catalytically active and deaminate targeted adenosines in dsRNA,[27] while ADAR3, which is expressed mainly in the brain, does not display any deaminase activity.[34] Humans express two isoforms of ADAR1 from two different promoters: a constitutively expressed ADAR1 with a molar mass of ∼110 kDa (ADAR1-p110) and an interferon-induced form with a molar mass of ∼150 kDa (ADAR1-p150).[35] All ADARs contain nuclear localization signals, thus delivering them to the nucleus where they can edit RNAs co- or post-transcriptionally.[36,37] However, the interferon-induced ADAR1 p150 contains a nuclear export signal (NES), allowing this isoform to distribute in both the nucleus and cytoplasm.[35] Cytoplasmic ADAR1 p150 plays an important role in regulating the interaction of dsRNA with the cytoplasmic dsRNA receptors MDA5 and PKR.[30,38,39]

Zα/Zβ DOMAIN

ADAR1 differs from ADAR2 and ADAR3 in two respects: first, ADAR1 contains one or two Zα-like domains for the p110 and p150 isoforms, respectively. The second distinct difference between ADAR1 and ADAR2/3 is the number of dsRNA binding domains (dsRBDs), which will be discussed in more detail below. Zα domains are small ∼65- to 70-residue domains that have been shown to bind to the left-handed double-stranded helical Z-DNA[40,41] and Z-RNA.[42] However, the second Zα-like domain in ADAR1 p150 and first in p110 is labeled Zβ because it reportedly lacks the ability to bind Z-DNA.[43,44] The only other known cellular proteins that contain a Zα domain are the mammalian Z-DNA-binding protein ZBP1 (previously known as DLM-1),[45] which also plays a role in innate immunity,[46] and the protein kinase PKZ found in fish.[47] Additionally, Zα domain proteins are also found in some viruses.[48] The function of the Zα domains in ADAR1 p150 is a bit enigmatic, but Z-DNA can transiently form upstream of an active RNA polymerase, possibly providing a platform for Zα binding of ADAR1 to facilitate editing of nascent RNA strands that fold into hairpins.[49,50] However, given that ADAR1 p150 isoform expression is regulated under a different promotor than the constitutively expressed p110, the function of Zα domain in ADAR1 p150, like ZBP1, may play a role in the innate immunity pathway.[45,51] Indeed, the ability of the Zα domains of ADAR1 and ZBP1 to bind Z-RNA is consistent with the hypothesis that ADAR1 p150 prevents binding of ZBP1 to immunogenic RNAs.[52,53]

Zα domains fold into a compact α+β structure called a winged helix-turn-helix (HTH) domain with a small three-helical bundle capped with a short two-stranded antiparallel β sheet (α1-α2-α3-β1-β2) (Figure 2). The crystal structure has been determined of human ADAR1 Zα domain complexed with Z-DNA (PDB: 1qbj, 2acj, and 3f21)[41,54,55] and with Z-RNA (PDB: 2gxb),[42] and the crystal structure of human ADAR1 Zβ has been determined (PDB: 1xmk).[56] Even though Zβ does not bind Z-DNA, both the Zα and Zβ structures are very similar in structure with a root-mean-squared deviation ranging from 0.79–1.1 Å for ∼53 equivalent Cα atoms, depending on the structure use for alignment (Figure 2A). The Zβ structure

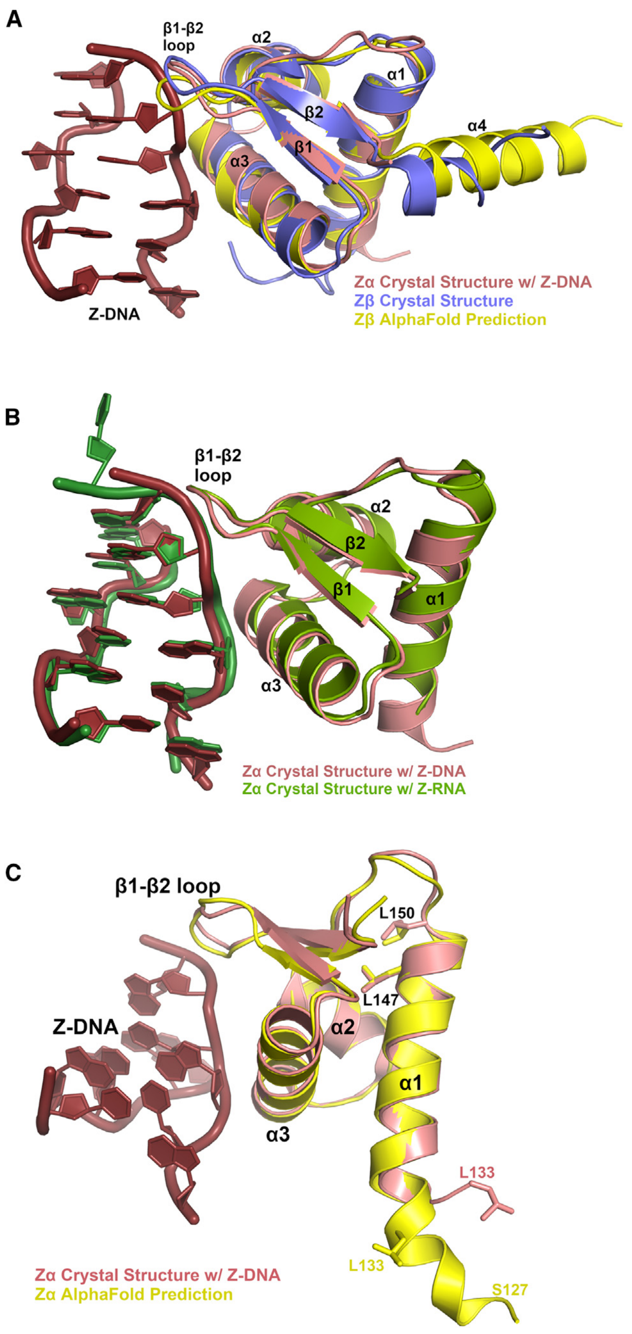

Figure 2. Structures of ADAR1 Z-alpha domains

(A) Crystal structures of human ADAR1 Zα domain bound to Z-DNA in salmon and ruby color, respectively (PDB: 3f21), superimposed onto the Zβ domain, slate blue (PDB: 1xmk). Also shown in yellow is the predicted Zβ domain from AlphaFold2. (B) Crystal structures of human ADAR1 Zα domain bound to Z-DNA in salmon and ruby color, respectively (PDB: 3f21), superimposed onto the ADAR1 Zα domain bound to Z-RNA (PDB: 2gxb), green and forest green color. (C) Crystal Structure of human ADAR1 Zα domain bound to Z-DNA in salmon and ruby color, respectively (PDB: 3f21), superimposed onto the ADAR1 Zα domain predicted by AlphaFold2 showing the extended helix α1 exhibiting all the leucines implicated in the nuclear export signal on the same face of helix α1.

has an additional α helix (α4) at the C-terminal end after β2. However, while sequence-based domain deciphering suggests that Zβ ends at residue 360, the AlphaFold2-predicted structure indicates that α4 may be extended to residue 371, where it makes additional hydrophobic and ionic interactions with residues in α1 (Figure 2A). For Zα, helices α2 and α3 and the β1-β2 loop mainly contact the zigzag phosphodiester backbone of the double helix, which likely provides the basis for specificity of the Z-conformation of either DNA or RNA (Figure 2B).

Interestingly, the NES of ADAR1 p150 lies within helix α1 of the Zα domain, which exports the p150 isoform into the cytoplasm. This helix contains leucines that defines a "leucine-rich" NES,[57] which is shuttled outside the nucleus through its recognition by the nuclear export receptor CRM1.[58] Mutating three of the leucines prohibits nuclear export.[59] However, based on the crystal structure, it appears the first leucine of this motif (Leu133) may not lie in helix α1 because sequence-based domain delineations suggests Zα begins at Leu133-Ile135, which guided the constructs used for crystallography, but an AlphaFold2 model[60] of this domain reveals that α1 of the Zα domain may extend further in the N-terminal direction, positioning all three leucines on the same face of helix α1 (Figure 2C). This N-terminal helical extension of α1 is not observed in the Zβ domain of ADAR1 (PDB: 1xmk), nor predicted by AlphaFold2, but it contains helix α4 at the C-terminal end of the domain.

dsRNA BINDING DOMAINS

All ADARs contain dsRBDs (sometimes referred to as dsRNA binding motifs) at the N-terminal side of the catalytic domain (Figure 1B). However, the number of dsRBDs varies based on the ADAR paralog and phylum of the organism. ADAR1 of vertebrates typically has three dsRBDs, while ADAR2 and the non-catalytic ADAR3 have two dsRBDs. Some invertebrate ADARs have a diverse number of dsRBDs, such as squid ADAR1, which is reported to have one dsRBD but an extended serine-rich sequence where the other dsRBDs are typically located,[61] while squid ADAR2 has an extra dsRBD.[62] There is evidence that ADAR from *Hydra vulgaris* may have five dsRBDs (unpublished data).

First identified in *Drosophila* Staufen, involved in mRNA localization, and PKR, a dsRNA-dependent protein kinase,[63,64] dsRBDs are found in over 100 proteins classified in at least nine families with diverse functions.[65] Some of the families include RNA helicases,[66] RNases,[67] DICER,[68] ribosomal proteins,[69] translation initiation factor kinases,[68] and ADARs.[70] The dsRBDs are small motifs consisting of 65–70 amino acids that typically bind double-helical A-form dsRNA in a sequence-independent manner.[71] The high-resolution three-dimensional structures of dsRBDs from human ADARs have been determined using nuclear magnetic resonance (NMR) and X-ray crystallography techniques, both alone and complexed with dsRNA.[72–76] However, the X-ray crystal structures of dsRBD2 bound to dsRNA are part of the larger construct that includes the deaminase domain of human ADAR2 bound to a larger fragment of RNA (discussed further below).[74,75]

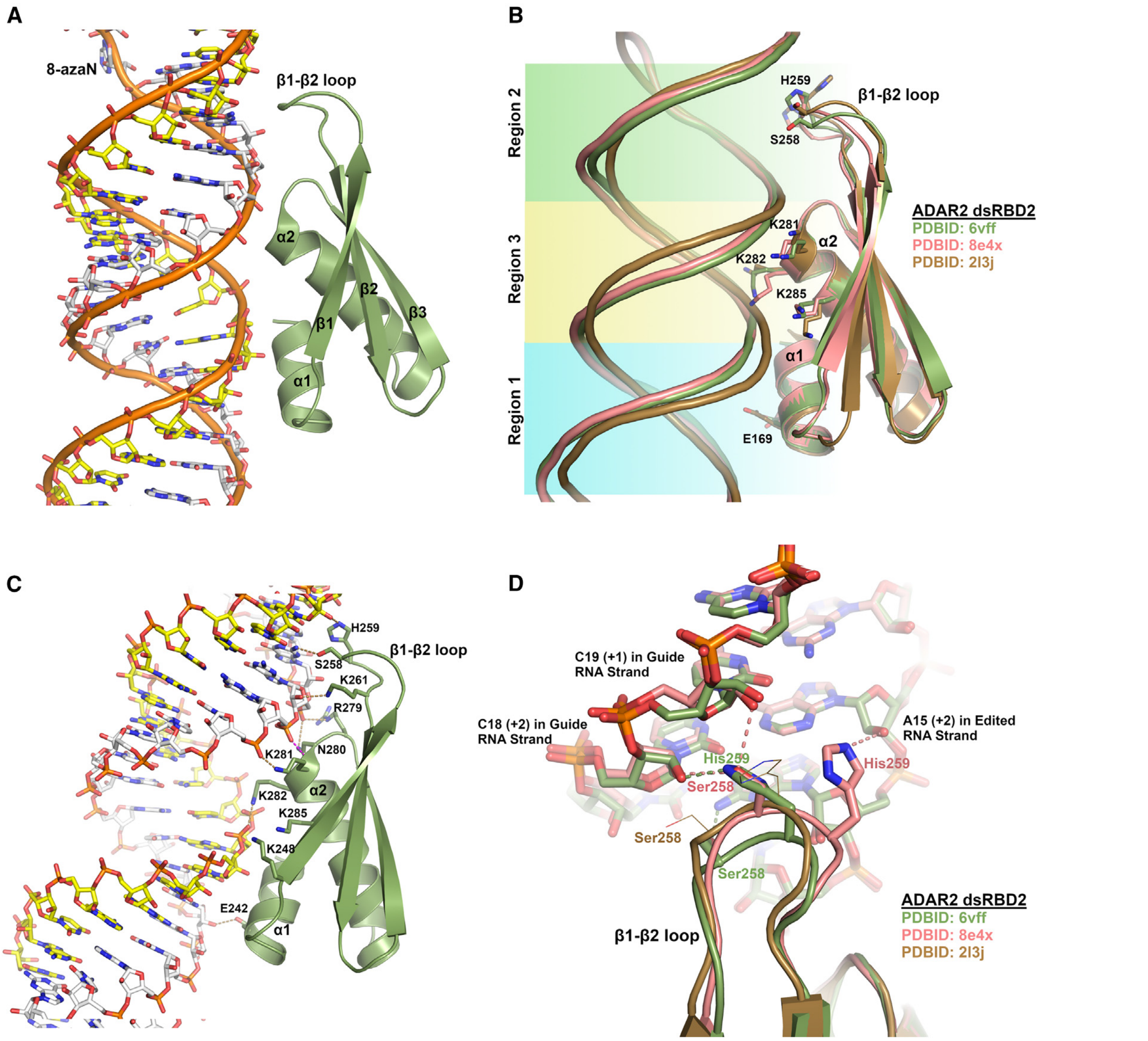

Figure 3. Structure of ADAR dsRBDs

(A) Overall fold of dsRBD from human ADAR2 bound to duplex RNA (PDB: 6vff). Conserved secondary structure elements of dsRBDs are labeled. For clarity, the deaminase domains of the ADAR2 dimer are omitted. The RNA strand that is edited by ADAR2 is shown with white-colored carbon atoms, with the 8-azaN labeled, which is flipped out of the duplex into the deaminase domain active site (not shown), and the non-edited RNA guide strand has yellow-colored carbons. (B) Comparison of some dsRBD structures bound to dsRNA. Residues corresponding to the three conserved regions that contact RNA are shown, Glu169 in helix α1 of region 1, His259 of β1-β2 loop of region 2, and the KKxxK motif in helix α2 of region 3. This figure also shows the conformational variability of region 2 in the β1-β2 loop among the different structures. (C) Detailed dsRBD-RNA interactions as seen in the ADAR2-R2D construct bound to dsRNA (PDB: 6vff). Shown in brown dashed lines are hydrogen bonds between RNA and dsRBD side chains. The magenta-colored dashed line is between the N-terminal dipole main-chain amide of α2 interacting with phosphate backbone. Lysines 285 and 248 don't make direct RNA contact but contribute an electrostatic potential. (D) Closeup view of β1-β2 loop's structural variability contacting the minor groove. Ser258 and His259 make different RNA contacts seen in different structures. Dash lines are colored to correspond to respective structure colors. These residues are not modeled to contact RNA in the NMR structure (PDB: 2l3j).

The dsRBD fold consists of a mixed α/β fold with a conserved α1-β1-β2-β3-α2 topology where helix α2 packs against the three-stranded antiparallel β sheet and helix α1 packs against helix α2. Helix α1 is shorter and impinges on the minor groove of the dsRNA but does not make any sequence-specific contacts (Figure 3A). There are three regions of the dsRBDs that directly contact one side of duplex RNA that spans two successive minor grooves and the intervening major groove[65,77] (Figure 3B). Region 1 comprises helix α1 that lies parallel to and contacts the minor groove and contains residues that interact with riboses and/or the phosphate backbone. However, in dsRBDs, there are only three highly conserved residues in region 1 (α1).[65] One highly conserved glutamate is found in this helix that usually

contacts a ribose 2′-hydroxyl oxygen. Another highly conserved residue in helix α1 is a leucine, two residues preceding the conserved glutamate, which packs in a hydrophobic pocket between α2 and the β sheet maintaining the dsRBD core fold. Finally, a highly conserve proline commences helix α1. Region 2 of dsRBDs that contact dsRNA is found in the β1-β2 loop. This loop bends in to contact the previous minor groove, relative to region 1, which is closer to the editing site in the ADAR2 structure.[74] Similar to region 1, this sequence is highly variable among the large family of dsRBDs, but a highly conserved histidine residue is found to interact with ribose 2′-hydoxyls.[72,74,75,77] Also highly conserved in this loop is a Gly-Pro motif two to three residues preceding the histidine. While the proline is not conserved in dsRBD2 of human ADAR2 for which there is a crystal structure, the Gly-Pro residues of other structures reveal they don't contact the RNA and are more solvent exposed.[72,77] The dipeptide sequence could facilitate proper β1-β2 loop conformation to contact RNA. Interestingly, the lack of proline in human ADAR2 dsRBD2 could allow for a more flexible β1-β2 loop, providing more adaptability and alternative RNA contacts (see below). The final region 3 of the dsRBDs that interacts with RNA is found at the N-terminal region of helix α2, which lies more perpendicular to the RNA helical axis. The N-terminal end of helix α2, which possesses the positive helix dipole moment seen in many phosphate-binding motifs[78] and some DNA binding HTH motifs,[79] abuts the phosphate backbone of the edited RNA strand where a phosphate makes a contact with the main-chain nitrogen at the start of helix α2 (Figure 3C). Additionally, this helix displays a highly conserved KKxxK motif whose lysine residues reside on the same face of helix α2 and point toward the dsRNA major groove between the two minor groove contacts defined by region 1 and 2. While not strictly conserved in all dsRBDs, the three lysine residues are conserved in all human ADAR dsRBDs, apart from the nonfunctional human ADAR3, where KKxxK is conserved in dsRBD1, but the sequence is KKxxR in dsRBD2. ADAR3 also conserves residues in region 1 and 2 implicated in RNA binding consistent with the observation that ADAR3 can bind to dsRNA structures.[34,80]

The structures of dsRBDs from ADAR2 have been determined, both alone[73] and in complex with dsRNA.[72,74,75] The only published structure of a dsRBD from ADAR1 is of the third dsRBD in the absence of RNA. This NMR structure revealed a short additional helix preceding α1, which is called α_N, and it contains part of the unusual "split" nuclear localization signal that flanks the dsRBD3 motif core.[76] This additional helix (α_N) juxtaposes the N-terminal and C-terminal residues flanking the dsRBD3, which are recognized by the nuclear import receptor transportin 1 for nuclear import.

Focusing on ADAR2's dsRBDs binding to RNA, Figure 3C shows potential contacts between dsRBD2 and RNA, as illustrated by dashed lines. All interactions, except one, are non-sequence-specific contacts that interact with either the phosphodiester backbone or 2′-OH of the ribose, emphasizing the dsRBD's ability to distinguish between of A-form dsRNA and B-form dsDNA. The one base-specific contact is the interaction between Ser258 of the β1-β2 loop, which hydrogen-bonds with the 2-amino group of guanosine 16 of the edited strand, three nucleotides downstream of the edited adenosine at position 13 (PDB: 6vff). However, this is likely not a sequence-defining contact because two other crystal structures of ADAR2 with dsRBD2 bound to RNA reveal slightly different conformations of the β1-β2 loop where Ser258 makes weak hydrogen bonds (∼3.3Å) to the 2′-OH of cytidine 19 of the non-edited RNA strand (PDB: 8eof and 8efx).[75] Comparing these three structures of the construct we call ADAR2-R2D, consisting of dsRBD2 and the deaminase domain (residues 235–701) bound to slightly different RNA substrates that vary in the nucleotide 5′ to the edited adenosine (PDB: 6vff, 8eof, and 8efx), reveals flexibility in the β1-β2 loop resulting in different RNA contacts between Ser258 and His259. His259 in two of the structures interacts with the 2′-OH of adenosine 15 of the edited strand, while in the other structure (PDB: 6vff) His259 flips across the minor groove and contacts the 2′-OH of cytidine 18 of the non-edited guide strand (Figure 3D). The NMR structure of rat ADAR2 dsRBD2 bound to RNA reveals that the equivalent histidine points away from the minor groove (PDB: 2l3j) (Figure 3D). Furthermore, while the histidine in the β1-β2 loop is highly conserved in all ADAR dsRBDs, it appears that Ser258 is only conserved in dsRBD2 of ADAR2, indicating this contact is not crucial to RNA binding. Finally, in the three crystal structures of ADAR2-R2D, the electron density of the dsRBD2 domain is weaker than the deaminase domain, suggesting that dsRBD2 binding to dsRNA may be a bit more tenuous and may bind in conformations "out of register" due to its ability to simply recognize A-form dsRNA in a sequence-independent manner, which can result in overall weaker electron density of this domain.

DEAMINASE DOMAIN STRUCTURE

All ADARs have a conserved catalytic deaminase domain that comprises the last ∼400 C-terminal residues. The first high-resolution structure of an ADAR deaminase domain comes from human ADAR2, which was determined in the absence of RNA (PDB: 1zy7).[81] The deaminase domain contains a seven-stranded mostly parallel β sheet core with the long α helix (α1) packed perpendicularly across one side of the β sheet and eight α helices on the other side of the β sheet core. The structure also revealed the expected zinc ion in the active site. The zinc ion, which is tetrahedrally coordinated, is ligated by two cysteine thiolates from Cys451 and Cys516 (human ADAR2 numbering) and the imidazole of His394. The fourth ligand is an ordered water molecule that when deprotonated to hydroxide is assumed to attack C6 of the adenine ring on the RNA and displaces the amino group in the deamination reaction (Figure 1A).

At first glance, it appears the ADARs don't resemble other RNA/DNA editing enzymes, but closer inspection reveals that there is some similarity to the APOBEC cytidine deaminases that can edit single-stranded RNA and/or DNA.[82] The APOBEC and ADAR deaminase domains structures share a similar five-stranded β sheet core together with two α helices that are involved in zinc binding.[2] Additionally, the ADARs do have a signature sequence motif found in APOBECs but with larger insertions. The sequence motif for ADARs is HXE-$X_{\sim 55}$-PC$X_{\sim 65}$C, where H and C are zinc ligands, and the E is the

active site glutamate residue essential in shuttling protons in the catalytic mechanism. Compared to the APOBEC signature sequence, ADARs have a large ∼55-residue insertion between the catalytic Glu and the first Cys zinc ligand, which is 25–30 residues in APOBECs. Additionally, there is a sizable ∼65-residue insertion between the two cysteine zinc ligands, which is typically only 1–4 residues in APOBECs. This second substantial insert in ADAR is noteworthy because it contains motifs essential for ADAR function and dsRNA binding. The insert contains the base-flipping loop, the dimerization helix, and the loop that defines RNA substrate selectivity differences between ADAR1 and ADAR2 in what we term the 5′ RNA binding loop (see below). This loop also contains a ligand for a second zinc binding site observed in ADAR1 but not in ADAR2.

This first ADAR structure was groundbreaking because it revealed for the first time the unexpected finding that ADARs require the cofactor inositol hexakisphosphate (IP_6) for protein folding and deaminase activity.[81] This report also demonstrated that IP_6 is required for the related eukaryotic adenosine deaminases that act on tRNA 1 (ADAT1) but not the eukaryotic ADAT2/ADAT3 heterodimer family of tRNA deaminases. The IP_6 molecule binds in the core of the ADAR deaminase domain, where eight, mostly invariant, lysine and arginine residues form ionic bonds to the six phosphates of IP_6. Using human ADAR2 numbering, Arg400, Arg401, Lys519, Lys662, and Lys690 are strictly conserved in ADAR1, ADAR2, and ADAR3 across many diverse species, including insect, fish, rodent, bovine, feline, amphibian, reptilian, avian, and primate ADARs. Lys672 is mostly conserved but is replaced by arginine in some amphibian ADAR1s and Arg522 invariant except in rodent ADAR3 where it is serine. The exact function of IP_6 in ADARs is a bit enigmatic. Given that this cofactor is buried in the deaminase core, it is essential for proper protein folding and activity.[81] However, while the center of the IP_6 is ∼14 Å from the active site zinc ion, the two cofactors are connected by an ionic bond relay employing the side chains of strictly conserved residues, Lys519-Asp392-Lys483 (human ADAR2 numbering). The amino group of Lys483 is 3.9 Å away from the active site zinc ion (diametrically opposite the catalytic water ligand) and 3.2 Å away from the Cys516 thiolate, one of the zinc ligands. It is interesting to highlight that a positively charged residue lies near the “backside” of the active site zinc ion (opposite the attacking water ligand) in many RNA/DNA editing APOBEC cytidine deaminases and the nucleotide cytidine deaminase (CDA) and adenosine deaminase (ADA). It is assumed that these positively charged residues partially neutralize the zinc ligand cysteine thiolates. Mutating the arginine residue near the zinc ion in CDA greatly reduced its V_{max}, but not K_m in cytidine deamination.[83] Therefore, the IP_6 moiety may indirectly influence ADAR activity through the conserved ionic bonding network to the active-site zinc ion, possibly affecting the pK_a of the zinc water ligand that attacks C6 of the adenine ring during catalysis.

ADAR2 DEAMINASE DOMAIN-dsRNA STRUCTURES

To assemble a stable ADAR-dsRNA complex required for structural studies, like X-ray crystallography, the nucleotide analog 8-azanebularine (8-azaN) was incorporated at the targeted adenosine position. This 8-azaN analog is expected to undergo the first hydration step of the ADAR mechanism (Figure 1A), but it is unable to complete the reaction because it lacks the 6-amino leaving group, thus mimicking the proposed high-energy intermediate state[84] and forming a stable tight-binding complex with nano-molar affinity.[28]

The first ADAR-dsRNA structure determined to high resolution consisted of the deaminase domain of human ADAR2 (ADAR2d) complexed with a duplex 23mer, with 8-azaN incorporated in the edited strand.[28] The structure revealed that the targeted adenosine analog, 8-azaN, is flipped out of the duplex RNA and inserts into the ADAR active site, where it interacts with the active zinc ion, catalytic glutamate (Glu396), and several other residues (Figure 4). The adenosine analog is pushed out of the duplex RNA by the base-flipping loop, which approaches the duplex RNA on minor groove face. The base-flipping loop contains the strictly conserved Gly-Glu-Gly sequence where the glutamate (Glu488) penetrates the duplex RNA flipping out the adenosine analog into the active site, whereupon Glu488 remains intercalated in the RNA where it makes two hydrogen bonds with the “orphaned” base opposite the flipped base. Glu488 is flanked by conserved glycines enabling the closest approach and deepest penetration possible by Glu488 to stabilize the flipped-base conformation for catalysis.

Remarkably, when this base-flipping Glu488 is mutated to a glutamine (E488Q), the resultant mutant displays higher deaminase activity,[85] and it binds to the 8-azaN containing duplex with higher affinity.[86] Structures of the hyperactive ADAR2d E488Q mutant complexed with 23-mer duplex RNAs revealed that these structures are nearly identical to the wild-type base-flipping loop[28] where Gln488 likewise inserts in the duplex RNA, flips out the targeted adenosine (8-azaN), and forms two contacts with the orphaned base cytidine (Figure 5A). Given that N3 of the cytosine ring is a hydrogen-bonding acceptor in the most stable tautomer, it is likely that the Gln488 amino group of the amide side chain hydrogen-bonds to N3 of the orphaned cytidine. However, for the wild-type base-flipping Glu488, at physiological pH, it is expected that the side chain can’t donate a hydrogen bond to N3, which may explain the longer contact distance (3.11 Å vs. 2.85Å, Glu488 vs. Gln488) (Figures 5B and 5C). Therefore, the neutral Gln488 capacity to intercalate in the minor groove and form a stronger interaction with the orphaned cytidine may explain why the ADAR2 E448Q mutant displays higher catalytic efficiency. These conclusions are also corroborated with pH studies on adenosine base-flipping and deaminase activity of wild-type ADAR2, which reveal that lower pH and protonation of Glu488 more readily flips out the adenosine and displays higher ADAR2 activity.[87,88]

These structures and pH analyses motivated us to seek nucleotide analogs that when substituted for cytidine at the orphan position, would interact more favorably with the wild-type Glu488 to increase ADAR’s editing efficiency. Specifically, a pyrimidine-size analog that can deliver a hydrogen-bonding donor at the N3 position while maintaining a hydrogen-bonding acceptor at the C2 exocyclic carbonyl oxygen to interact with the deprotonated glutamate 488

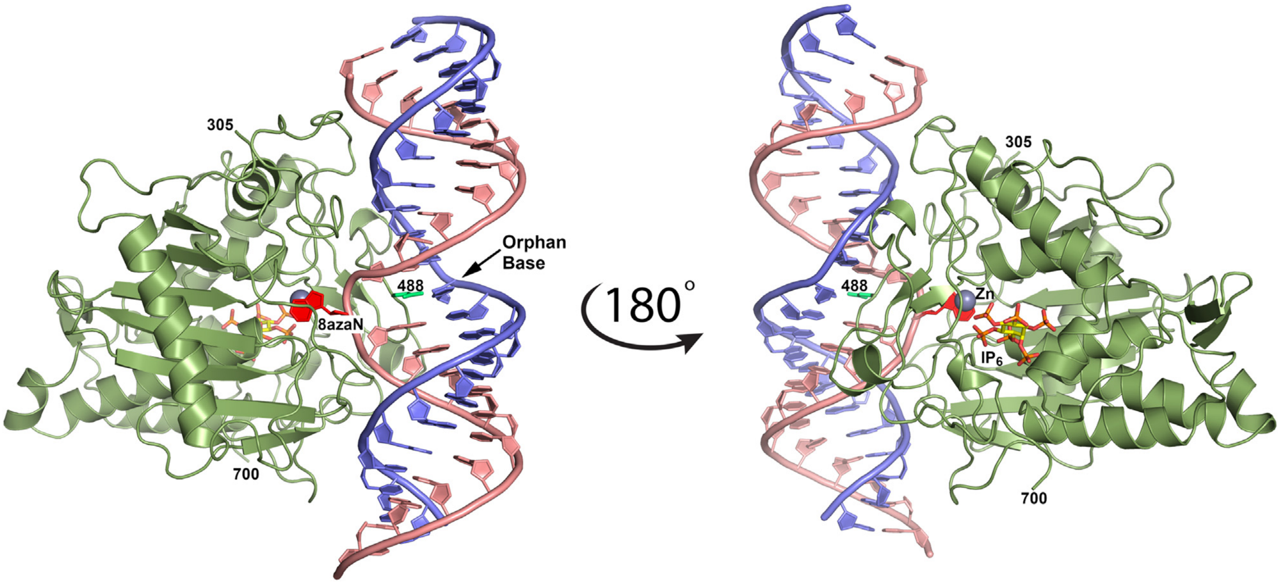

Figure 4. Crystal structure of human ADAR2 deaminase domain complexed with dsRNA 23-mer

Edited RNA strand is salmon colored. The adenosine analog 8-azaN (red) is flipped out into the active site where it interacts with the zinc ion (gray sphere). The base-flipping residue 488 is shown in bright green and IP_6 as sticks with yellow-colored carbon atoms.

could serve this task to enhance editing. 6-Amino-5-nitro-3-(1′-β-D-2′-deoxyribofuranosyl)-2-(1H)-pyridinone (Benner's base dZ), which was first synthesized for artificially expanded genetic information systems,[89] is a pyrimidine-sized analog that contains a hydrogen bond donor at the N3 equivalent position and provides a hydrogen bond acceptor at the exocyclic O2 position to interact with Glu488 main-chain nitrogen (Figure 5D). Indeed, wild-type ADAR2 displayed higher editing efficiency when dZ is substituted in the guide-strand orphan position, observed in biochemical assays and in cultured cells, compared to cytidine.[87] The high-resolution crystal structure of human ADAR2d bound to a duplex RNA with the dZ analog at the orphan position confirmed the tighter interaction with wild-type base-flipping Glu488. Interestingly the side chain carboxylate of Glu488 makes bifurcated hydrogen bonds to the ring nitrogen (N1 in dZ base numbering) and the exocyclic amine (Figure 5D). This contact distance is shorter in dZ compared to the cytidine (2.83 Å vs. 3.11Å and 3.37Å) suggesting a stronger interaction. Additionally, the orphan dZ exocyclic carbonyl oxygen makes a shorter contact to the main-chain nitrogen of Glu488 (2.67Å vs. 2.93Å) (Figures 5C and 5D). Furthermore, density functional theory calculations indicate that the partial charge of the *donor-donor-acceptor* face of the dZ base is more positive than the *donor-acceptor-acceptor* face of dC, suggesting that the electrostatic interaction between the partial positive face of dZ and the carboxylate of Glu488 may provide additional favorable interaction energy.[87]

ADARs SEQUENCE CONTEXT PREFERENCES

The structures of ADAR2d with duplex RNA also provide a structural basis for ADAR's editing sequence preferences, such as the preference to edit adenosine between a 5′-U and a 3′-G.[90,91] The structure reveals that the 2-amino group of guanosine, which projects into the minor groove of dsRNA, defines this sequence preference for both flanking nucleotides, positively and negatively for the 3′-G and 5′-U sequence preference, respectively. The structure shows that the 2-amino group of the 3′-G of the editing strand forms a sequence-specific hydrogen bond to the main-chain carbonyl oxygen of Ser486 in the base-flipping loop (Figure 6A). If the 2-amino group of the 3′-G is removed by replacing the guanosine with inosine, which can still base pair with the complementary cytidine, the catalytic deamination rate decreases by ∼50%.[28]

A 2-amino group on the 5′ side of the targeted adenosine has a negative impact on editing. Modeling in a guanosine at the 5′ side of the edited adenosine demonstrated that the projection of the 2-amino group in the minor groove would result in the nitrogen atom coming within 3 Å of the Cα of Gly489, resulting in a van der Waals clash from hydrogens on both atoms (Figure 6A). Equivalently, adenosines with 5′-C neighbors are not efficiently edited by ADARs, because the complementary base-paired guanosine would also have a 2-amino group in a van der Waals clash with Gly489 (Figure 6A). This is validated when the adenosine complementary to the preferred 5′-U is replaced with 2-amino purine (2AP), which is still capable of base pairing with the 5′-U. However, this nucleotide analog delivers a 2-amino group in the minor groove that can clash with Gly489, resulting in deaminase rates of the 2AP:U base pair similar to a G:C base pair.[28]

Despite the bias against ADARs efficiently editing adenosines with a 5′-G, it has been shown that if the 5′-G is mismatched with another purine in the −1 position of the guide strand, the rate of editing is greatly enhanced.[75,92] To understand the structural basis of these

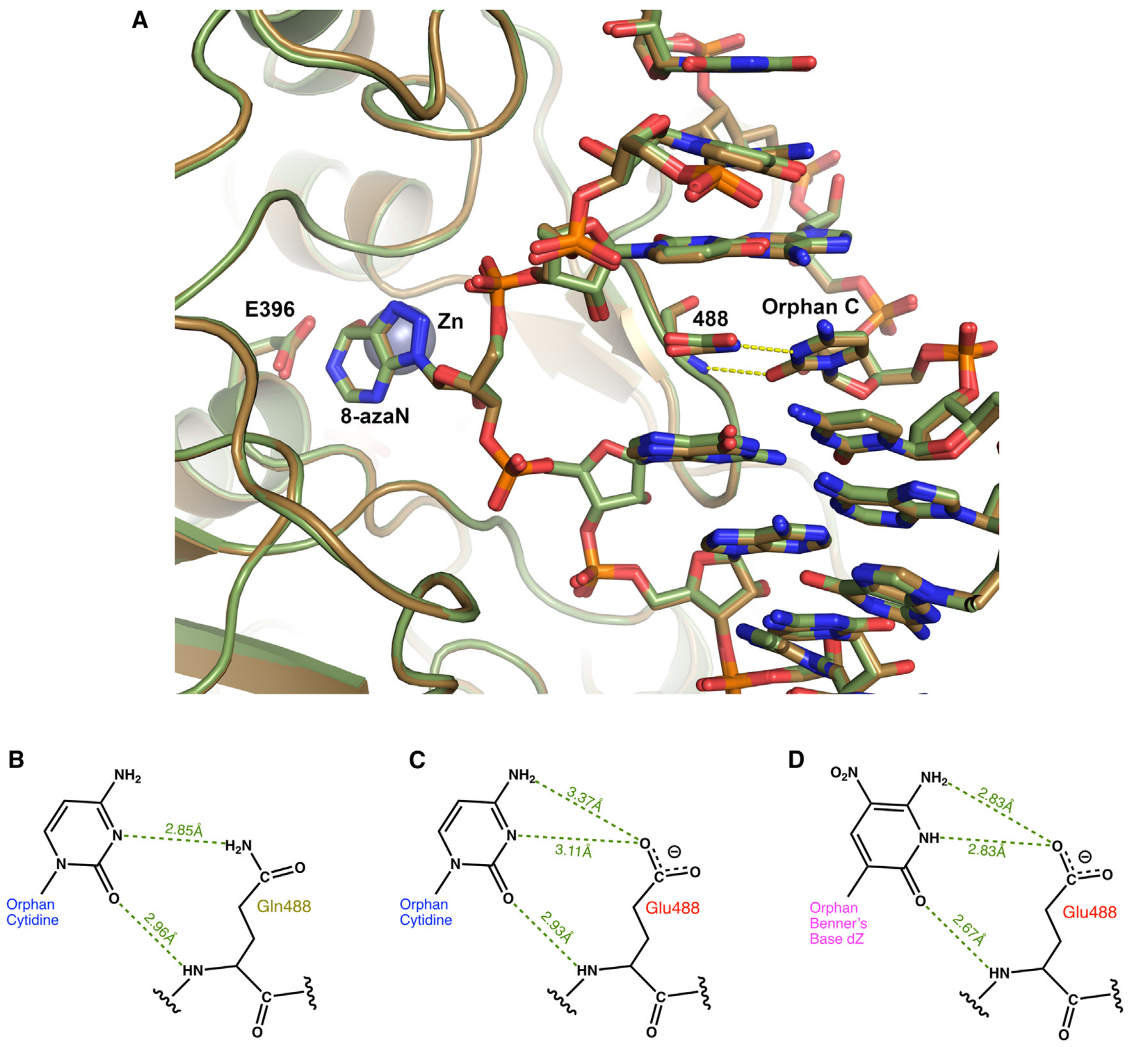

Figure 5. Closeup view of base-flipping loop and orphan base interactions

(A) View of the base-flipping loop and active site of human ADAR2 deaminase domain of the wild-type enzyme (PDB: 5hp3, in green), superimposed on the hyperactive mutant (PDB: 5ed1, brown). The base-flipping residue 488 makes two potential hydrogen bonds with the orphaned cytidine base. Side chain of 488 contacts N3 of the cytosine ring, while the exocyclic 2-carbonyl oxygen hydrogen-bonds to the main-chain amide nitrogen of 488. (B) Interactions between the base-flipping residue 488 and the orphan base opposite the flipped adenosine. Hyperactive E488Q makes two hydrogen bonds with orphan cytidine with the main chain and side chain. (C) Wild-type Glu488 makes a main-chain hydrogen bond, but the deprotonated side chain distance increases to 3.11 Å to N3 of cytidine. (D) Substituting Benner's base dZ in the orphan position creates tighter interactions to base-flipping Glu488 compared to cytidine. Two or three hydrogen bonds can form with wild-type Glu488. Side-chain carboxyl oxygen of Glu488 can hydrogen-bond with either the dZ N1 nitrogen (dZ numbering) or the exocyclic amino group. Distances shown are between heavy atoms (not hydrogen).

findings, we determined the structure of human ADAR2 complexed with RNA that contains a G:G mismatch at the 5′-G.[75] This structure revealed that the clash between the 5′-G 2-amino group and the base-flipping loop can be mitigated by inverting the 5′-G base into the *syn* conformation, which is stabilized by a Hoogsteen base-pairing interaction with the −1 G of the guide strand, which resides in the canonical *anti* conformation (Figure 6B). However, a guanosine in the −1 position of the guide strand still presents a 2-amino group into the minor groove that pushes the base-flipping Gly489 about 1 Å toward the flipped-out 8-azaN. Eliminating this 2-amino group by substituting in a 3-deaza-2′-deoxyadenosine at the −1 position of the guide strand still promotes the 5′G to adopt a *syn* conformation, but more importantly, it eliminates the push on the base-flipping loop Gly489 and increases the ADAR editing rate even more than the G:G mismatch.[75] 3-Deaza-adenosine is more effective than adenosine likely because the pK_a of N1 of the 3-deaza adenosine is higher (6.8) compared to adenosine (3.7), allowing N1 to furnish a hydrogen bond donor to the Hoogsteen edge accepting N7 of the *syn* 5′-G (Figure 6B).[75] This hydrogen-bonding pattern causes the bases to slide slightly (compared to G:G), allowing the base-flipping loop to shift, which results in the swinging of the 8-azaN phosphate to the ideal position seen with the preferred 5′-U structure (Figure 6B).

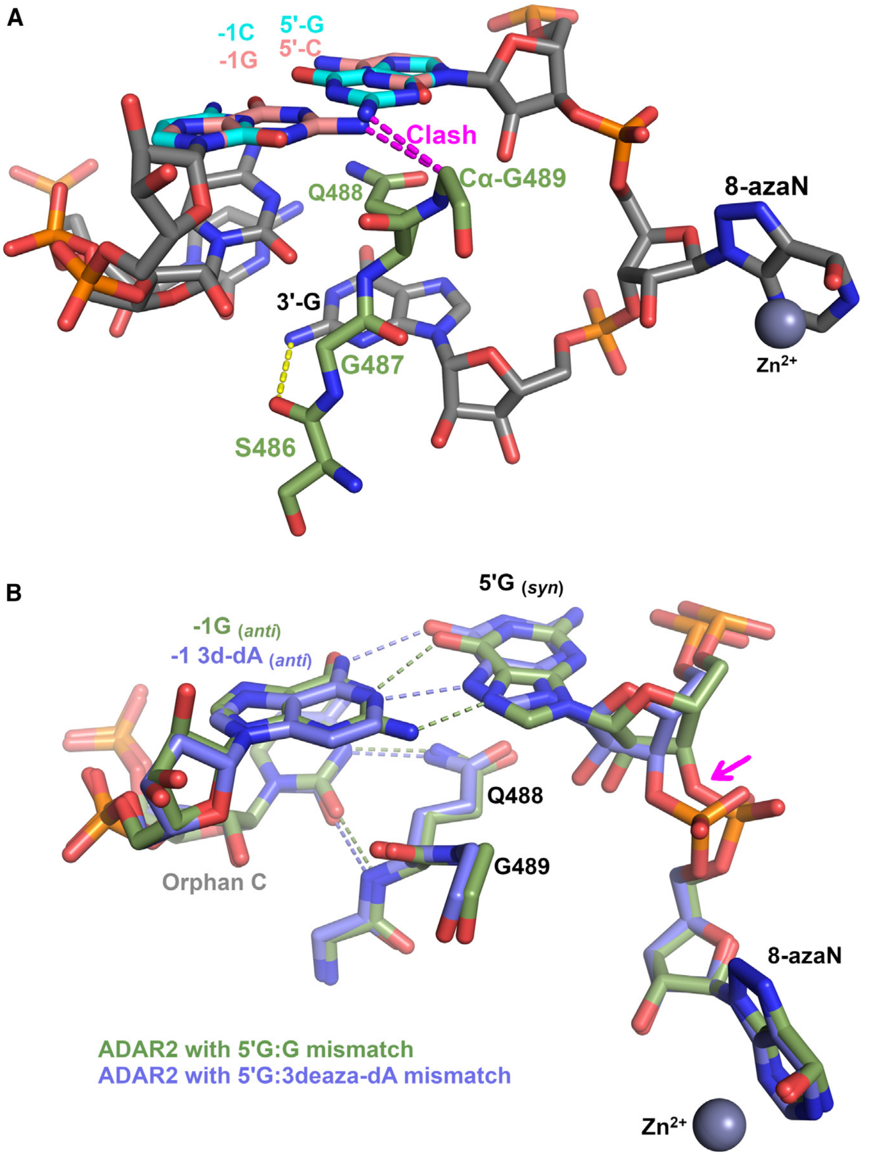

Figure 6. Structural basis for ADAR's sequence preference of 5′-UAG-3′

(A) Based on the crystal structure of the ADAR2d-RNA complex, a 5′-G or 5′-C was modeled with the complementary base pair. Shown is the base-flipping loop in green-colored carbon atoms, with part of the dsRNA (gray-colored carbons). The 5′-U:A base pair was modeled to 5′-G:C (cyan) and 5′-C:G (salmon) base pair illustrating a clash between the guanosine exocyclic 2-amino group with Gly489 (magenta dashes). Also shown is the interaction between the preferred 3′-G's 2-amino group hydrogen bonding with the main-chain carbonyl oxygen of Ser486. (B) Structures of human ADAR2 with a 5′-G in the edited strand and purine nucleotides at the −1 position of the guide RNA strand, either guanosine (green) (PDB: 8e0f) or 3-deaza-2′-deoxyadenosine (blue) (PDB: 8e4x). Both guide-strand purines create a mismatch with the 5′-G, causing this guanosine to flip into the *syn* conformation and forming a Hoogsteen base pair (dashes) with the −1 purine of the guide strand. The colors of dashed lines correspond to Hoogsteen hydrogen bonding of the two different structures as shown. Magenta arrow shows movement of 8-azaN 5′-phosphate to a conformation seen in the ideal 5′U:A structure.

ADAR DEAMINASE DOMAIN-RNA INTERACTION DETAILS

The structure of the ADAR2 deaminase domain complexed with the 23-mer dsRNA also reveals other protein-RNA contacts that are important for A-form duplex recognition and efficient editing activity. However, outside the base-flipping Glu488 interaction with the orphan base, the 3′-G 2-amino contact with Ser486, and catalytic Glu396 interacting with the flipped adenosine analog, all other protein-RNA contacts are sequence-independent interactions with the RNA ribose or phosphodiester backbone. The majority of ADAR2 residues that contact RNA are found in the large insert between the two cysteine residues that ligate the catalytic zinc (Figure 7). In ADARs, this insert is ∼65–70 residues long compared with 1–4 residues seen in the APOBECs, ADAs, and CDAs. This large insert contains the base-flipping loop and the 5′-RNA binding loop, which was disordered in the RNA-free ADAR2 structure.[81] The base-flipping loop that contacts the RNA on the minor groove is shown in brown in Figure 7 and defines ADAR2's nearest neighbor preferences. The 5′-RNA binding loop (magenta color in Figure 7), which contacts RNA on the 5′ side of the edited adenosine, contains four arginine residues (455, 474, 477, and 481) that contact the phosphate backbone of both the edited and guide RNA strand. The 5′-RNA binding loop also contains Asn473 whose side chain and main chain contact RNA. Of the four arginines in the 5′-binding loop, only Arg481 is strictly conserved in all ADARs (1, 2, and 3). The other arginines 455, 474, and 477 are conserved in ADAR2 and ADAR3, but not ADAR1, where they are replaced by Ala, Asn, and Gln, respectively. Asn473 is also conserved in ADAR2 and 3 but is a glutamate in ADAR1. These 5′-binding loop sequence variations, together with a longer 5′-loop in ADAR1, help to define the different sequence specificity in RNA substrate preferences between ADAR1 and ADAR2.[93] It is also noteworthy to point out that in ADAR1, this 5′-RNA binding loop contains His988 that ligates a second zinc site not seen in ADAR2.[94] Therefore, zinc may dictate ADAR1's sequence specificity through altered 5′-loop conformations with the loading and unloading of a second zinc ion.

While the majority of the dsRNA structure resides in the expected A-form helical conformation, a perturbation is observed at the editing

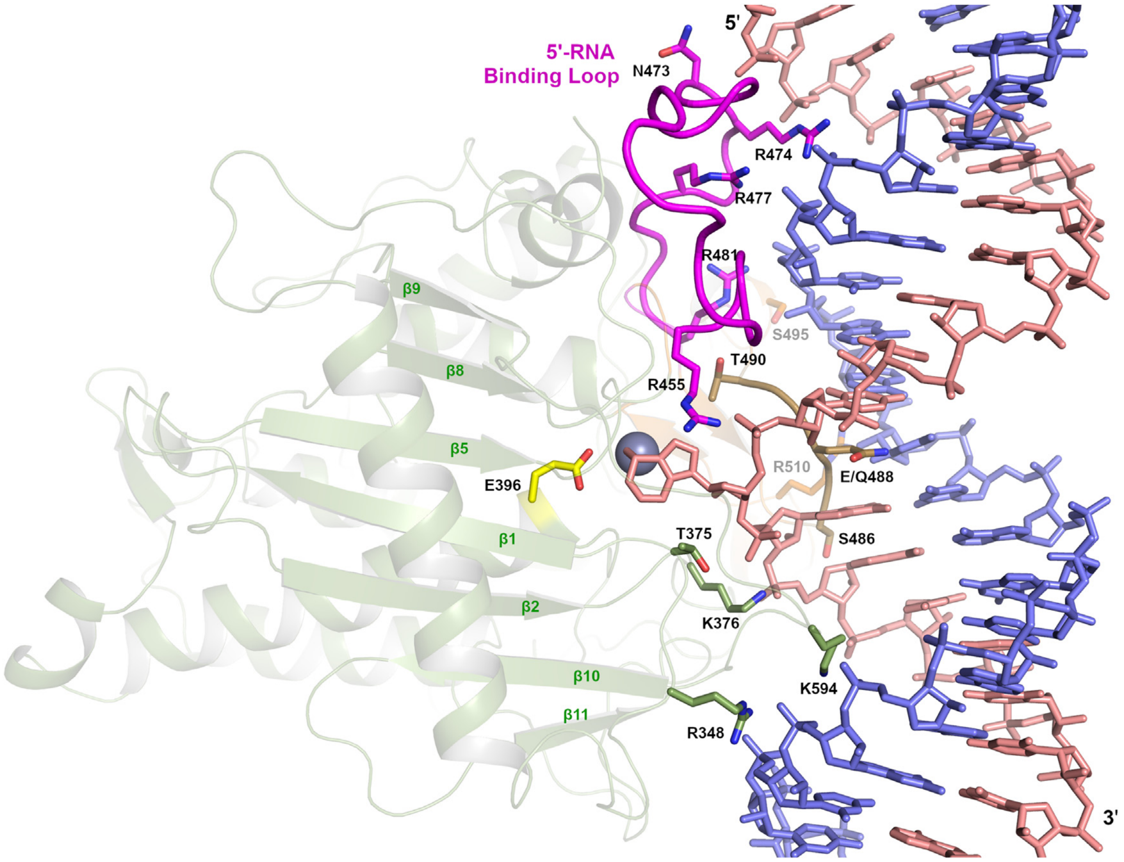

Figure 7. ADAR2d-RNA interactions

The ADAR2 deaminase domain shown in semi-transparent cartoon in green displaying the residues that interact with RNA, shown with stick side chains. The base-flipping loop that contacts the minor groove and orphan base is shown in brown. The 5′-RNA binding loop is shown in magenta color. The remainder of the C-$X_{\sim 65}$-C insert is shown in orange. Residues that contact the 3′-end of the edited strand are shown with green-colored carbon atoms. Catalytic Glu396 is in yellow, and the edited RNA strand is in salmon color, with the flipped-out 8-azaN.

site. The ADAR2 base-flipping residue 488 requires a deep penetration from the minor groove to flip out the adenosine and hydrogen bond to the orphaned base. This close approach necessitates a shift from the A-form helix resulting in the "sliding" of the base pair at the 5′ side of the editing site away from the ADAR protein, which induces the widening of the RNA major groove opposite the editing site.[28] This "sliding" of the 5′ side base pair causes both ribose sugar puckers to adopt a C2′-*endo* conformation, which is normally observed in the B-form helical configuration. Both the 5′-U (5′ to the flipped-out 8-azaN) and its complementary adenosine on the guide strand in the −1 position (see Figure S1 for numbering) reside in this B-form ribose sugar pucker (Figure S1). In addition to the intercalation of residue 488 and interaction with the orphaned base, its side chain also hydrogen-bonds to the 5′-U ribose 2′-OH, stabilizing this C2′-*endo* sugar pucker. It is significant to point out that the C2′-*endo* conformation in the guide-strand −1 position is necessary for efficient editing because substituting a locked nucleic acid, which is locked in the C3′-*endo* conformation, abolishes editing, whereas replacing this nucleotide with a deoxyribose enhances editing.[95] It is also interesting to note that the only other ribose that adopts a C2′-*endo* conformation is the flipped-out adenosine analog where the main-chain carbonyl of Thr375 induces this conformation by hydrogen bonding to the 2′-OH of 8-azaN. The C2′-*endo* conformation of the flipped-out base's ribose likely places the base in an ideal position for the nucleophilic attack of the zinc-bound water at C6. Therefore, the ribose C2′-*endo* sugar pucker in these three nucleotides helps to define ideal substrate conformation, which can be exploited to help enhance or suppress RNA editing as desired.

STRUCTURE OF ADAR DEAMINASE DOMAIN + dsRBD2 COMPLEXED WITH RNA

To better understand RNA recognition and the structural influence of a dsRBD binding to dsRNA in the context of the catalytic deaminase domain, we determined the crystal structure of human ADAR2 construct called ADAR2-R2D, which consists of residues 215 to the C-terminal residue 701.[74] This construct comprises the second dsRBD (dsRBD2) and the deaminase domain. The structure was determined with a longer fragment of the Gli1 dsRNA consisting of

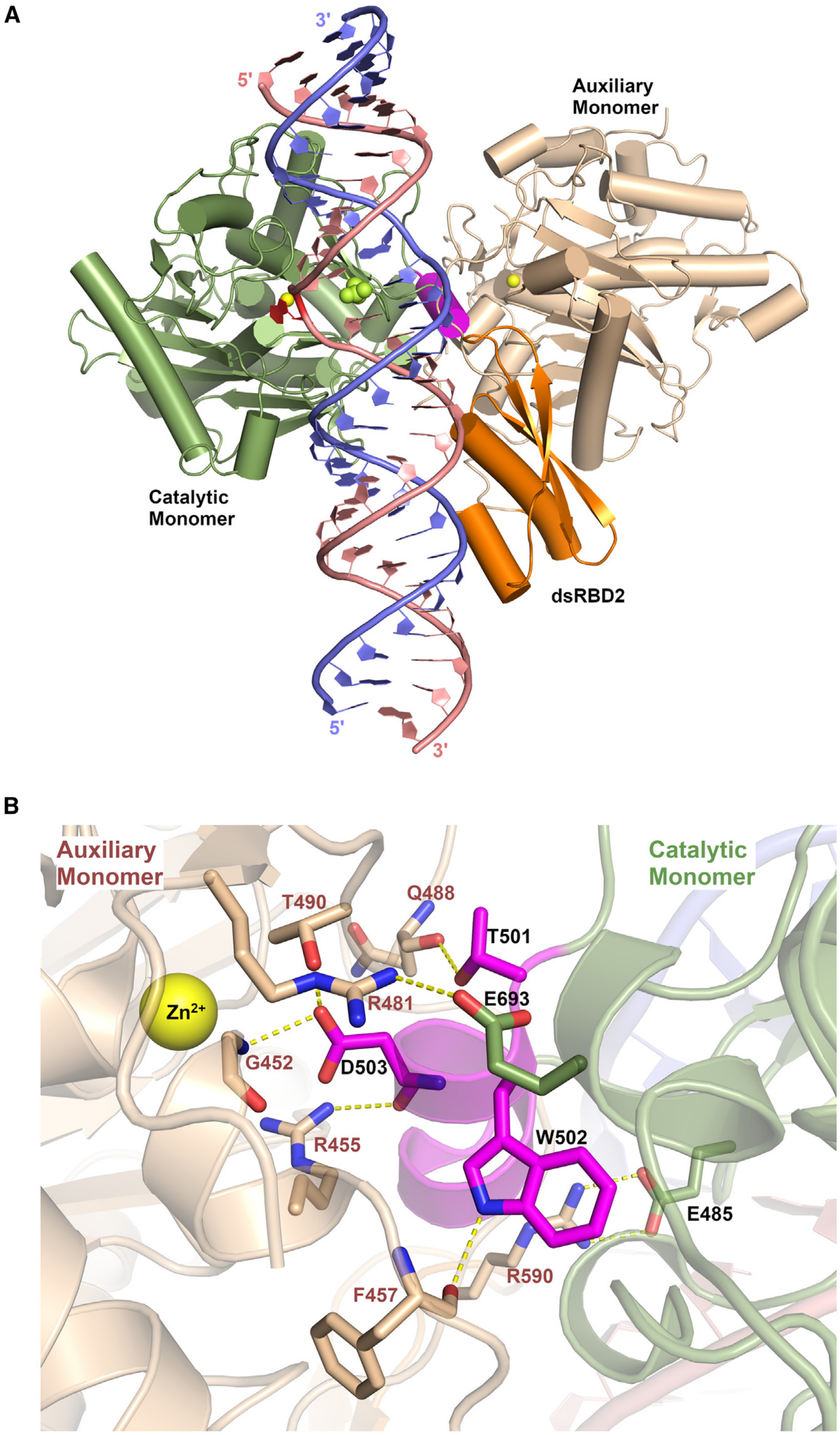

Figure 8. Crystal structure of human ADAR2-R2D complexed with a 32-mer RNA

(A) Catalytic monomer is shown in green, and auxiliary monomer in light tan, whose dsRBD2 is orange, binds to the 3′ end of the dsRNA. The flipped-out 8-azaN analog is shown in red, and the base-flipping residue 488 is shown in lime-green spheres. The dimerization helix from the catalytic monomer is shown in magenta color, which binds to the active site of the auxiliary monomer, and zinc ions are in yellow spheres. (B) Zoomed view of the deaminase domain dimerization contacts. Catalytic monomer (green) dimerization helix (magenta) binds in the active-site pocket of the auxiliary monomer (tan). Residues that participate in dimerization contacts are shown as sticks and hydrogen bonds with yellow dashed lines.

a 32-mer duplex containing the 8-azaN analog at the targeted adenosine base to trap a stable intermediate complex with the 8-azaN flipped into the deaminase active site. Based on footprinting protection experiments, the 32-mer duplex was extended toward the 3′ direction (relative to the edited adenosine) compared to the 23-mer dsRNA used in the deaminase-RNA structures detailed above.[74]

The crystal structure of the ADAR2-R2D complexed with the 32-mer dsRNA surprisingly revealed the enzyme assembled onto the dsRNA as an asymmetric homodimer where one monomer's active site binds the flipped-out 8-azaN, as seen previously (called the catalytic monomer), plus a second monomer's active site binds to a short alpha helix of the catalytic monomer (Figure 8A). This second "auxiliary" monomer's deaminase domain does not directly engage the RNA duplex, but its dsRNA binding domain is seen to bind to the duplex RNA on the 3′ side of the flipped-out 8-azaN, where in interacts with both strands of RNA in a manner similar to other dsRBD-RNA structures. The dsRBD2 of the catalytic monomer is completely disordered in the crystal structure, suggesting it does not bind tightly or may bind dynamically or nonspecifically to the RNA.

All three regions of the auxiliary monomer's dsRBD2 contact dsRNA 3′ to the edited base. Specifically, region 2 (β1-β2 loop) contacts nucleotides as close as the +1 position in the guide strand and nucleotides +3 and +4 of the edited strand. (Numbering is relative to orphan base and flipped base, which is 0 in each strand, and positive numbers extend to the 5′ direction of the guide strand or 3′ direction of the edited strand.) Region 2 of the dsRBD2 (N-terminal end of helix α2) contacts the phosphate backbone of nucleotides +4, +5, and +6 of the edited strand and +12 of the guide strand. The first two lysines of the conserved KKxxK signature sequence found in all ADAR dsRBDs

are involved in the phosphate contacts, while the third lysine points toward the major groove to contribute to the overall positive electrostatic potential. Finally, the conserved glutamate in region 1 (helix α1) contacts the 2′-OH of nucleotide +14 in the edited strand. Additionally, the α1 N-terminal main-chain nitrogen contacts the 2′-OH of the +12 nucleotide in the guide strand.

The deaminase domains assemble into an asymmetric homodimer that is mediated by interactions between the dimerization helix of the catalytic monomer and the active site of the auxiliary monomer (Figure 8B). The dimerization helix contains a highly conserved TWDG motif, where the side chains of Thr501, Trp502, and Asp503 interact with the auxiliary dimer's active site. Specifically, Thr501 hydrogen bonds with main-chain carbonyl oxygen of Gln488 in the auxiliary monomer (which is the base-flipping residue in catalytic monomer). The indole nitrogen of Trp502 interacts with the main-chain carbonyl oxygen of Phe457, and Asp503 of the dimerization helix interacts with both the main-chain amide nitrogen of Gly452 and the side chain of Thr490 (Figure 8B). Additionally, the carboxylate group of Asp503 wedges into an electrostatic cavity created by Arg481 and Arg455 of the auxiliary monomer's active site. Gly504 of the TWDG motif is conserved in the dimerization helix because it makes a close contact with conserved Gly489 of the auxiliary monomer, which in the catalytic monomer is adjacent to the base-flipping 488. In addition to these dimerization helix-mediated contacts, the dimer is stabilized by the ionic interactions between Arg481 and Arg590 of the auxiliary monomer and Glu693 and Glu485 of the catalytic monomer, respectively (Figure 8B). It is significant to highlight that all the residues from the auxiliary monomer that facilitate dimer contacts also play a critical role in forging RNA contacts in the catalytic monomer. Therefore, these residues adopt a dual functionality: protein dimerization in the auxiliary monomer and RNA binding in the catalytic monomer.

ADAR DIMERIZATION

The asymmetric homodimerization of ADAR2 on its RNA substrate is important for efficient RNA editing. Disrupting this dimerization through mutations of the conserved TWDG motif in the dimerization helix can greatly diminish editing activity on many RNA substrates, especially mutating Asp503, which lodges in the positive potential pocket defined by Arg455 and Arg481.[74] The D503A mutation in ADAR2 disrupts the dimer and greatly reduces ADAR2 editing efficiency both in biochemical assays and in HEK293T cells.[74] More importantly, the same mutation in ADAR1 D1023A also reduces RNA editing in HEK293T cells, which suggests that ADAR1 also exists in a similar asymmetric homodimerization complex upon binding RNA substrates.

Additional validation that this asymmetric homodimer readily forms and is biologically functional comes from other ADAR2 structures. The original RNA-free crystal structure of human ADAR2 deaminase domain also assembles into a similar asymmetric dimer when inspecting the crystal packing (Figure S2).[81] Furthermore, AlphaFold2 predicts that both ADAR1 and ADAR2 assemble into similar asymmetric homodimers (Figure S2).[60] Additionally, AlphaFold2 predicts that the deaminase domain of the nonfunctional ADAR3 also may organize into a similar dimer, but one monomer is rotated roughly 32° compared to the ADAR1 and ADAR2 dimers.

Curiously, if AlphaFold2 is used to predict possible heterodimer interactions between human ADAR1 and ADAR2, it also predicts a similar arrangement of deaminase domain monomers, where the heterodimer contact is also mediated by the conserved dimerization helix (Figure S2). In fact, AlphaFold2 predicts two types of ADAR1-ADAR2 heterodimers, one where ADAR1d serves in the catalytic position and ADAR2d in the auxiliary position and the opposite arrangement with ADAR2d in the catalytic location and ADAR1d in the auxiliary site. This advances the possibility of ADAR1-ADAR2 heterodimerization, which might be used for regulation or expanding the RNA binding capabilities to edit additional RNA transcripts that might not be efficiently edited by homodimers. Remarkably, AlphaFold2 also predicts heterodimers between ADAR3-ADAR1 and ADAR3-ADAR2, also in similar dimeric arrangements (Figure S2). However, of significance is that unlike the ADAR1-ADAR2 heterodimer predictions where either deaminase domain can reside in either monomer position (catalytic or auxiliary), the ADAR3 heterodimer predictions always position the nonfunctional ADAR3 domain in the catalytic monomer position where its dimerization helix occupies the active site of ADAR1 or ADAR2. Formation of such complexes would explain how ADAR3 might inhibit ADAR1 and ADAR2 activity, which has been reported.[34,80,96]

ADAR1 STRUCTURE CONJECTURES

At the time of writing, there are no published structures of ADAR1 deaminase domain from any organism, either alone or complexed with RNA substrate. However, some ADAR1 structural insight can be gleaned from the homologous ADAR2 structures and the predicted AlphaFold2 structure. As outlined above, AlphaFold2 does predict a potential deaminase domain asymmetric homodimer as observed in human ADAR2 where the conserved dimerization helix of one deaminase domain binds to the active site of another monomer. Therefore, it is expected that ADAR1 likely binds to dsRNA substrates as asymmetric homodimers as observed in ADAR2.[97]

The deaminase domains of human ADAR1 and ADAR2 are 37.1% identical (54.6% similar) in amino acid sequence. As expected, residues important for function are strictly conserved, like the Gly-Glu-Gly base-flipping loop, the catalytic zinc ligands, the catalytic glutamate involved in shuttling protons in the adenosine to inosine mechanism, and the positively charged residues that bind the IP_6 molecule. However, notable differences are observed in the 5′ RNA binding loop that play important roles in distinguishing the selectivity between ADAR1 and ADAR2. This loop (residues 455–477 in human ADAR2) was disordered in the original RNA-free ADAR2 deaminase domain structure but is seen in the RNA-bound structure because it contacts the dsRNA substrate due to several protein-RNA interactions. This loop, which is 5 residues longer in ADAR1, only preserves a conserved phenylalanine and proline between the two ADAR

sequences, but the functional significance is uncertain since these residues don't contact RNA or appear to be structurally crucial. Interestingly, residues in this loop that interact with RNA substrate are conserved in the ADAR2 family but not in the ADAR1 family, suggesting this loop can contribute to the distinct RNA substrate specificity. In fact, when these loops are interchanged between ADAR1 and ADAR2, RNA substrate preferences are also swapped. Specifically, substituting in the human ADAR2 5′-RNA binding loop for the equivalent loop in ADAR1 produces a chimeric protein that displays ADAR2-like deaminase selectivity.[93]

Another interesting feature of the 5′-RNA binding loop in ADAR1 is that it contains a ligand for a second zinc binding site. High-throughput mutagenesis, molecular modeling, and metal ion analysis identified a second zinc binding site in ADAR1 distinct from the catalytic zinc ion.[94] Specifically, mutating human ADAR1 Cys1081, Cys1082, His1103, and His988 resulted in loss of the second zinc binding, as determined by inductively coupled plasma mass spectrometry (ICP-MS), and no observable activity on tested RNA substrates. One of the ligands for the second zinc site, His988, resides in the 5′-RNA binding loop for ADAR1. The AlphaFold2 structure of human ADAR1 indeed predicted a structure with all four residues congregating together with a vacancy that can accommodate a zinc ion that is tetrahedrally coordinated by the four residues (Figure S3). Superposition of the predicted ADAR1 deaminase domain onto the ADAR2-R2D structure with bound RNA reveals that the ADAR1 5′-RNA binding loop is pulled away from the RNA because of its interaction with the second zinc site. Sequence alignment of these 5′-binding loops reveals that they contain several conserved positively charged residues. While the sequences align within each ADAR family, the positively charged residues don't align between the ADAR1 and ADAR2 families, both in primary sequence and in structural space. In ADAR2, most residues are found to interact with RNA, while the positively charged conserved residues in the ADAR1 5′-loop are predicted to lie too far from a dsRNA substrate modeled from the superposition of the ADAR2-R2D structure. Therefore, ADAR1's conserved cationic residues in the 5′-RNA binding loop cannot interact with RNA in this ADAR2-based model (Figure S3). Therefore, ADAR1 may bind and edit RNAs with more bent structures or structures with large bulges on the 5′ side of the edited adenosine. Conversely, one can speculate that ADAR1's second zinc site, whose binding affinity has not been reported, might modulate RNA binding. If the His988 zinc ligand is liberated, e.g., by protonation of His988, this could release the 5′-RNA binding loop from the zinc tether, allowing the loop to flip up, thus enabling some of the conserved positively charged residues to interact with more linear duplex RNAs.

The residues in ADAR2 that interact with RNA on the 3′ side of the targeted adenosine are partially conserved between ADAR2 and ADAR1. Arg348 in ADAR2, which ion pairs with the phosphate of G12 in the guide RNA oligo, maps to Gly865 in ADAR1. However, based on the AlphaFold predicted structure, ADAR1 Lys1122 fills the void from the absent side chain of Gly865 and can interact with the phosphate backbone. This Lys1122 is indeed conserved in ADAR1 from diverse species, suggesting it likely contacts the RNA substrate. Lys376 of ADAR2, which interacts with 3′-phosphate of the flipped-out 8-azaN, corresponds to Arg892 of ADAR1, which is conserved in the ADAR1 family. This suggests this residue, like Lys376 of ADAR2, is also important in stabilizing the flipped-out adenosine in the active site. Finally, Lys594 of ADAR2 points in the major groove toward the 3′-phosphate of the flipped 8-azaN and is conserved in the ADAR1 family (Lys1120). This lysine is part of an RQXGK motif conserved in ADAR1 and ADAR2. Arg1116 of ADAR1 in this motif also points in toward the major groove near Lys1120 and may interact with RNA phosphate backbone. Also noteworthy is the conservation of glycine in this motif, which makes a close approach to the RNA phosphodiester backbone in ADAR2-RNA complexes. Mutating this residue to alanine in ADAR2 (G593A) decreases deamination rate over 10-fold and larger side chains almost 100-fold.[28]

CONCLUSIONS

ADARs have attracted much attention recently for the capabilities to bind dsRNA and recode specific adenosines to inosine, which is "read" as guanosine, effectively making an A-to-G mutation in RNA. Therefore, there is much enthusiasm in the prospects of redeploying endogenous ADARs to selectively correct disease-causing mutations on RNA by delivering a short guide RNA oligomer to target the disease-causing mutation, forming a duplex RNA framework that serves as a substrate for ADAR in site-directed RNA editing (SDRE).[98–100] High-resolution structures of ADARs bound to diverse RNA substrates would advance this field significantly by helping to direct guide RNA modifications that can both increase editing efficiency and stabilize the RNA oligomers within the cell. Several proofs of this principle have already been demonstrated in using the high-resolution structures to improve editing. One example was motivated by the NMR structure of ADAR2's dsRBDs bound to the R/G editing site of GluR2.[72] This structure led to linking the RNA hairpin of this R/G site to a guide RNA to serve as an ADAR recruitment domain to help direct endogenous ADAR2 to the desired editing site.[101] Another example is the use of ADAR2-RNA structures to guide the design of nucleotide analogs that can more favorably interact with the glutamate residue present in the base-flipping loop, thus increasing editing efficiency.[87] The ADAR2-RNA structures were also used to recognize noncanonical ribose sugar puckers at specific positions and for harnessing this information to help guide nucleotide analog placement to either increase or decrease editing at specific adenosines.[95] The structures also provided an explanation for ADAR's nearest neighbor preferences, which was exploited to enable editing at adenosine with an adjacent 5′ guanosine by incorporating bases in the guide RNA that induce the 5′ guanosine to flip into the *syn* conformation.[75]

All these structure-guided optimizations and modulations have been focused on ADAR2 because there are no published structures of ADAR1 from any organism. This is because ADAR1 has proven to be a bit recalcitrant to efficient heterologous ectopic expression and purification needed for many biophysical studies. Yet ADAR1 has garnered more interest in SDRE because it is expressed in most tissues, necessitating the need for high-resolution structures of this

important enzyme. Furthermore, there are no structures known of full-length ADARs, because the size and flexibility between the modular domains proves challenging in crystallization. However, given the recent cryoelectron microscopy advancements and the requirement for less protein and concentrations compared to crystallization, it is only a matter of time before many more ADAR structures will reveal a clearer picture of ADAR functionality.

SUPPLEMENTAL INFORMATION

Supplemental information can be found online at https://doi.org/10.1016/j.omtn.2024.102284.

ACKNOWLEDGMENTS

A.J.F. is supported by grants from the Department of Defense (W81XWH-22-1-0893), National Institutes of Health (R01 GM149799-01), and the United States Department of Agriculture (CA-D-MCB-2629-H). P.A.B acknowledges support from the National Institutes of Health (R35GM141907), the Rett Syndrome Research Trust, and ProQR Therapeutics. A.J.F. and P.A.B are jointly supported by the National Science Foundation (MCB-2315296).

AUTHOR CONTRIBUTIONS

A.J.F. and P.A.B. wrote the manuscript. A.J.F. created the figures.

DECLARATION OF INTERESTS

There authors declare no competing interests.

REFERENCES

1. Grosjean, H. (2005). Modification and editing of RNA: historical overview and important facts to remember. In Fine-tuning of RNA Functions by Modification and Editing, H. Grosjean, ed. (Springer Berlin Heidelberg), pp. 1–22. https://doi.org/10.1007/b106848.
2. Fisher, A.J., and Beal, P.A. (2018). Structural basis for eukaryotic mRNA modification. Curr. Opin. Struct. Biol. *53*, 59–68. https://doi.org/10.1016/j.sbi.2018.05.003.
3. Ontiveros, R.J., Stoute, J., and Liu, K.F. (2019). The chemical diversity of RNA modifications. Biochem. J. *476*, 1227–1245. https://doi.org/10.1042/BCJ20180445.
4. McCown, P.J., Ruszkowska, A., Kunkler, C.N., Breger, K., Hulewicz, J.P., Wang, M.C., Springer, N.A., and Brown, J.A. (2020). Naturally occurring modified ribonucleosides. Wiley interdisciplinary reviews. RNA *11*, e1595. https://doi.org/10.1002/wrna.1595.
5. Boccaletto, P., Stefaniak, F., Ray, A., Cappannini, A., Mukherjee, S., Purta, E., Kurkowska, M., Shirvanizadeh, N., Destefanis, E., Groza, P., et al. (2022). MODOMICS: a database of RNA modification pathways. 2021 update. Nucleic Acids Res. *50*, D231–D235. https://doi.org/10.1093/nar/gkab1083.
6. Boo, S.H., and Kim, Y.K. (2020). The emerging role of RNA modifications in the regulation of mRNA stability. Exp. Mol. Med. *52*, 400–408. https://doi.org/10.1038/s12276-020-0407-z.
7. Wilkinson, E., Cui, Y.H., and He, Y.Y. (2022). Roles of RNA Modifications in Diverse Cellular Functions. Front. Cell Dev. Biol. *10*, 828683. https://doi.org/10.3389/fcell.2022.828683.
8. Sarin, L.P., and Leidel, S.A. (2014). Modify or die?–RNA modification defects in metazoans. RNA Biol. *11*, 1555–1567. https://doi.org/10.4161/15476286.2014.992279.
9. Jonkhout, N., Tran, J., Smith, M.A., Schonrock, N., Mattick, J.S., and Novoa, E.M. (2017). The RNA modification landscape in human disease. RNA *23*, 1754–1769. https://doi.org/10.1261/rna.063503.117.
10. Slotkin, W., and Nishikura, K. (2013). Adenosine-to-inosine RNA editing and human disease. Genome Med. *5*, 105. https://doi.org/10.1186/gm508.
11. Motorin, Y., and Helm, M. (2022). RNA nucleotide methylation: 2021 update. Wiley interdisciplinary reviews. RNA *13*, e1691. https://doi.org/10.1002/wrna.1691.
12. Zhou, Y., Kong, Y., Fan, W., Tao, T., Xiao, Q., Li, N., and Zhu, X. (2020). Principles of RNA methylation and their implications for biology and medicine. Biomed. Pharmacother. *131*, 110731. https://doi.org/10.1016/j.biopha.2020.110731.
13. Flamand, M.N., Tegowski, M., and Meyer, K.D. (2023). The Proteins of mRNA Modification: Writers, Readers, and Erasers. Annu. Rev. Biochem. *92*, 145–173. https://doi.org/10.1146/annurev-biochem-052521-035330.
14. Duan, Y., Dou, S., Zhang, H., Wu, C., Wu, M., and Lu, J. (2018). Linkage of A-to-I RNA Editing in Metazoans and the Impact on Genome Evolution. Mol. Biol. Evol. *35*, 132–148. https://doi.org/10.1093/molbev/msx274.
15. Zhang, P., Zhu, Y., Guo, Q., Li, J., Zhan, X., Yu, H., Xie, N., Tan, H., Lundholm, N., Garcia-Cuetos, L., et al. (2023). On the origin and evolution of RNA editing in metazoans. Cell Rep. *42*, 112112. https://doi.org/10.1016/j.celrep.2023.112112.
16. Bass, B.L., and Weintraub, H. (1987). A developmentally regulated activity that unwinds RNA duplexes. Cell *48*, 607–613. https://doi.org/10.1016/0092-8674(87)90239-x.
17. de Reuver, R., and Maelfait, J. (2024). Novel insights into double-stranded RNA-mediated immunopathology. Nature reviews. Immunology *24*, 235–249. https://doi.org/10.1038/s41577-023-00940-3.
18. Eisenberg, E., and Levanon, E.Y. (2018). A-to-I RNA editing - immune protector and transcriptome diversifier. Nat. Rev. Genet. *19*, 473–490. https://doi.org/10.1038/s41576-018-0006-1.
19. Pestal, K., Funk, C.C., Snyder, J.M., Price, N.D., Treuting, P.M., and Stetson, D.B. (2015). Isoforms of RNA-Editing Enzyme ADAR1 Independently Control Nucleic Acid Sensor MDA5-Driven Autoimmunity and Multi-organ Development. Immunity *43*, 933–944. https://doi.org/10.1016/j.immuni.2015.11.001.
20. Rueter, S.M., Dawson, T.R., and Emeson, R.B. (1999). Regulation of alternative splicing by RNA editing. Nature *399*, 75–80. https://doi.org/10.1038/19992.
21. Solomon, O., Oren, S., Safran, M., Deshet-Unger, N., Akiva, P., Jacob-Hirsch, J., Cesarkas, K., Kabesa, R., Amariglio, N., Unger, R., et al. (2013). Global regulation of alternative splicing by adenosine deaminase acting on RNA (ADAR). RNA *19*, 591–604. https://doi.org/10.1261/rna.038042.112.
22. Eisenberg, E. (2021). Proteome Diversification by RNA Editing. Methods Mol. Biol. *2181*, 229–251. https://doi.org/10.1007/978-1-0716-0787-9_14.
23. Melcher, T., Maas, S., Herb, A., Sprengel, R., Seeburg, P.H., and Higuchi, M. (1996). A mammalian RNA editing enzyme. Nature *379*, 460–464. https://doi.org/10.1038/379460a0.
24. Brusa, R., Zimmermann, F., Koh, D.S., Feldmeyer, D., Gass, P., Seeburg, P.H., and Sprengel, R. (1995). Early-onset epilepsy and postnatal lethality associated with an editing-deficient GluR-B allele in mice. Science *270*, 1677–1680. https://doi.org/10.1126/science.270.5242.1677.
25. Nishikura, K. (2016). A-to-I editing of coding and non-coding RNAs by ADARs. Nat. Rev. Mol. Cell Biol. *17*, 83–96. https://doi.org/10.1038/nrm.2015.4.
26. Gabay, O., Shoshan, Y., Kopel, E., Ben-Zvi, U., Mann, T.D., Bressler, N., Cohen-Fultheim, R., Schaffer, A.A., Roth, S.H., Tzur, Z., et al. (2022). Landscape of adenosine-to-inosine RNA recoding across human tissues. Nat. Commun. *13*, 1184. https://doi.org/10.1038/s41467-022-28841-4.
27. Wang, Y., Zheng, Y., and Beal, P.A. (2017). Adenosine Deaminases That Act on RNA (ADARs). In The Enzymes, G. Chanfreau, ed. (Academic Press), pp. 215–268. https://doi.org/10.1016/bs.enz.2017.03.006.
28. Matthews, M.M., Thomas, J.M., Zheng, Y., Tran, K., Phelps, K.J., Scott, A.I., Havel, J., Fisher, A.J., and Beal, P.A. (2016). Structures of human ADAR2 bound to dsRNA reveal base-flipping mechanism and basis for site selectivity. Nat. Struct. Mol. Biol. *23*, 426–433. https://doi.org/10.1038/nsmb.3203.
29. Hartner, J.C., Schmittwolf, C., Kispert, A., Müller, A.M., Higuchi, M., and Seeburg, P.H. (2004). Liver disintegration in the mouse embryo caused by deficiency in the RNA-editing enzyme ADAR1. J. Biol. Chem. *279*, 4894–4902. https://doi.org/10.1074/jbc.M311347200.
30. Liddicoat, B.J., Piskol, R., Chalk, A.M., Ramaswami, G., Higuchi, M., Hartner, J.C., Li, J.B., Seeburg, P.H., and Walkley, C.R. (2015). RNA editing by ADAR1 prevents

MDA5 sensing of endogenous dsRNA as nonself. Science *349*, 1115–1120. https://doi.org/10.1126/science.aac7049.

31. Higuchi, M., Maas, S., Single, F.N., Hartner, J., Rozov, A., Burnashev, N., Feldmeyer, D., Sprengel, R., and Seeburg, P.H. (2000). Point mutation in an AMPA receptor gene rescues lethality in mice deficient in the RNA-editing enzyme ADAR2. Nature *406*, 78–81. https://doi.org/10.1038/35017558.

32. Duan, Y., Tang, X., and Lu, J. (2022). Evolutionary driving forces of A-to-I editing in metazoans. Wiley Interdiscip. Rev. RNA *13*, e1666. https://doi.org/10.1002/wrna.1666.

33. Savva, Y.A., Rieder, L.E., and Reenan, R.A. (2012). The ADAR protein family. Genome Biol. *13*, 252. https://doi.org/10.1186/gb-2012-13-12-252.

34. Chen, C.X., Cho, D.S., Wang, Q., Lai, F., Carter, K.C., and Nishikura, K. (2000). A third member of the RNA-specific adenosine deaminase gene family, ADAR3, contains both single- and double-stranded RNA binding domains. RNA *6*, 755–767. https://doi.org/10.1017/s1355838200000170.

35. Patterson, J.B., and Samuel, C.E. (1995). Expression and regulation by interferon of a double-stranded-RNA-specific adenosine deaminase from human cells: evidence for two forms of the deaminase. Mol. Cell Biol. *15*, 5376–5388. https://doi.org/10.1128/MCB.15.10.5376.

36. Rodriguez, J., Menet, J.S., and Rosbash, M. (2012). Nascent-seq indicates widespread cotranscriptional RNA editing in Drosophila. Mol. Cell *47*, 27–37. https://doi.org/10.1016/j.molcel.2012.05.002.

37. Licht, K., Kapoor, U., Amman, F., Picardi, E., Martin, D., Bajad, P., and Jantsch, M.F. (2019). A high resolution A-to-I editing map in the mouse identifies editing events controlled by pre-mRNA splicing. Genome Res. *29*, 1453–1463. https://doi.org/10.1101/gr.242636.118.

38. Mannion, N.M., Greenwood, S.M., Young, R., Cox, S., Brindle, J., Read, D., Nellåker, C., Vesely, C., Ponting, C.P., McLaughlin, P.J., et al. (2014). The RNA-editing enzyme ADAR1 controls innate immune responses to RNA. Cell Rep. *9*, 1482–1494. https://doi.org/10.1016/j.celrep.2014.10.041.

39. Chung, H., Calis, J.J.A., Wu, X., Sun, T., Yu, Y., Sarbanes, S.L., Dao Thi, V.L., Shilvock, A.R., Hoffmann, H.H., Rosenberg, B.R., and Rice, C.M. (2018). Human ADAR1 Prevents Endogenous RNA from Triggering Translational Shutdown. Cell *172*, 811–824.e14. https://doi.org/10.1016/j.cell.2017.12.038.

40. Herbert, A., Alfken, J., Kim, Y.G., Mian, I.S., Nishikura, K., and Rich, A. (1997). A Z-DNA binding domain present in the human editing enzyme, double-stranded RNA adenosine deaminase. Proc. Natl. Acad. Sci. USA *94*, 8421–8426. https://doi.org/10.1073/pnas.94.16.8421.

41. Schwartz, T., Rould, M.A., Lowenhaupt, K., Herbert, A., and Rich, A. (1999). Crystal structure of the Zalpha domain of the human editing enzyme ADAR1 bound to left-handed Z-DNA. Science *284*, 1841–1845. https://doi.org/10.1126/science.284.5421.1841.

42. Placido, D., Brown, B.A., 2nd, Lowenhaupt, K., Rich, A., and Athanasiadis, A. (2007). A left-handed RNA double helix bound by the Z alpha domain of the RNA-editing enzyme ADAR1. Structure *15*, 395–404. https://doi.org/10.1016/j.str.2007.03.001.

43. Schwartz, T., Lowenhaupt, K., Kim, Y.G., Li, L., Brown, B.A., 2nd, Herbert, A., and Rich, A. (1999). Proteolytic dissection of Zab, the Z-DNA-binding domain of human ADAR1. J. Biol. Chem. *274*, 2899–2906. https://doi.org/10.1074/jbc.274.5.2899.

44. Kim, Y.G., Muralinath, M., Brandt, T., Pearcy, M., Hauns, K., Lowenhaupt, K., Jacobs, B.L., and Rich, A. (2003). A role for Z-DNA binding in vaccinia virus pathogenesis. Proc. Natl. Acad. Sci. USA *100*, 6974–6979. https://doi.org/10.1073/pnas.0431131100.

45. Karki, R., and Kanneganti, T.D. (2023). ADAR1 and ZBP1 in innate immunity, cell death, and disease. Trends Immunol. *44*, 201–216. https://doi.org/10.1016/j.it.2023.01.001.

46. Zhong, Y., Zhong, X., Qiao, L., Wu, H., Liu, C., and Zhang, T. (2023). Zalpha domain proteins mediate the immune response. Front. Immunol. *14*, 1241694. https://doi.org/10.3389/fimmu.2023.1241694.

47. Rothenburg, S., Deigendesch, N., Dittmar, K., Koch-Nolte, F., Haag, F., Lowenhaupt, K., and Rich, A. (2005). A PKR-like eukaryotic initiation factor 2alpha kinase from zebrafish contains Z-DNA binding domains instead of dsRNA binding domains. Proc. Natl. Acad. Sci. USA *102*, 1602–1607. https://doi.org/10.1073/pnas.0408714102.

48. Quyen, D.V., Ha, S.C., Lowenhaupt, K., Rich, A., Kim, K.K., and Kim, Y.G. (2007). Characterization of DNA-binding activity of Z alpha domains from poxviruses and the importance of the beta-wing regions in converting B-DNA to Z-DNA. Nucleic Acids Res. *35*, 7714–7720. https://doi.org/10.1093/nar/gkm748.

49. Herbert, A., and Rich, A. (1996). The biology of left-handed Z-DNA. J. Biol. Chem. *271*, 11595–11598. https://doi.org/10.1074/jbc.271.20.11595.

50. Peck, L.J., Nordheim, A., Rich, A., and Wang, J.C. (1982). Flipping of cloned d(pCpG)n.d(pCpG)n DNA sequences from right- to left-handed helical structure by salt, Co(III), or negative supercoiling. Proc. Natl. Acad. Sci. USA *79*, 4560–4564. https://doi.org/10.1073/pnas.79.15.4560.

51. Guo, X., Liu, S., Sheng, Y., Zenati, M., Billiar, T., Herbert, A., and Wang, Q. (2023). ADAR1 Zalpha domain P195A mutation activates the MDA5-dependent RNA-sensing signaling pathway in brain without decreasing overall RNA editing. Cell Rep. *42*, 112733. https://doi.org/10.1016/j.celrep.2023.112733.

52. Zhang, T., Yin, C., Fedorov, A., Qiao, L., Bao, H., Beknazarov, N., Wang, S., Gautam, A., Williams, R.M., Crawford, J.C., et al. (2022). ADAR1 masks the cancer immunotherapeutic promise of ZBP1-driven necroptosis. Nature *606*, 594–602. https://doi.org/10.1038/s41586-022-04753-7.

53. Nichols, P.J., Krall, J.B., Henen, M.A., Vögeli, B., and Vicens, Q. (2023). Z-RNA biology: a central role in the innate immune response? RNA *29*, 273–281. https://doi.org/10.1261/rna.079429.122.

54. Ha, S.C., Lowenhaupt, K., Rich, A., Kim, Y.G., and Kim, K.K. (2005). Crystal structure of a junction between B-DNA and Z-DNA reveals two extruded bases. Nature *437*, 1183–1186. https://doi.org/10.1038/nature04088.

55. Ha, S.C., Choi, J., Hwang, H.Y., Rich, A., Kim, Y.G., and Kim, K.K. (2009). The structures of non-CG-repeat Z-DNAs co-crystallized with the Z-DNA-binding domain, hZ alpha(ADAR1). Nucleic Acids Res. *37*, 629–637. https://doi.org/10.1093/nar/gkn976.

56. Athanasiadis, A., Placido, D., Maas, S., Brown, B.A., 2nd, Lowenhaupt, K., and Rich, A. (2005). The crystal structure of the Zbeta domain of the RNA-editing enzyme ADAR1 reveals distinct conserved surfaces among Z-domains. J. Mol. Biol. *351*, 496–507. https://doi.org/10.1016/j.jmb.2005.06.028.

57. Kutay, U., and Güttinger, S. (2005). Leucine-rich nuclear-export signals: born to be weak. Trends Cell Biol. *15*, 121–124. https://doi.org/10.1016/j.tcb.2005.01.005.

58. Poulsen, H., Nilsson, J., Damgaard, C.K., Egebjerg, J., and Kjems, J. (2001). CRM1 mediates the export of ADAR1 through a nuclear export signal within the Z-DNA binding domain. Mol. Cell Biol. *21*, 7862–7871. https://doi.org/10.1128/MCB.21.22.7862-7871.2001.

59. Strehblow, A., Hallegger, M., and Jantsch, M.F. (2002). Nucleocytoplasmic distribution of human RNA-editing enzyme ADAR1 is modulated by double-stranded RNA-binding domains, a leucine-rich export signal, and a putative dimerization domain. Mol. Biol. Cell *13*, 3822–3835. https://doi.org/10.1091/mbc.e02-03-0161.

60. Jumper, J., Evans, R., Pritzel, A., Green, T., Figurnov, M., Ronneberger, O., Tunyasuvunakool, K., Bates, R., Žídek, A., Potapenko, A., et al. (2021). Highly accurate protein structure prediction with AlphaFold. Nature *596*, 583–589. https://doi.org/10.1038/s41586-021-03819-2.

61. Vallecillo-Viejo, I.C., Voss, G., Albertin, C.B., Liscovitch-Brauer, N., Eisenberg, E., and Rosenthal, J.J.C. (2023). Squid express conserved ADAR orthologs that possess novel features. Front. Genome Ed. *5*, 1181713. https://doi.org/10.3389/fgeed.2023.1181713.

62. Palavicini, J.P., O'Connell, M.A., and Rosenthal, J.J.C. (2009). An extra double-stranded RNA binding domain confers high activity to a squid RNA editing enzyme. RNA *15*, 1208–1218. https://doi.org/10.1261/rna.1471209.

63. St Johnston, D., Brown, N.H., Gall, J.G., and Jantsch, M. (1992). A conserved double-stranded RNA-binding domain. Proc. Natl. Acad. Sci. USA *89*, 10979–10983. https://doi.org/10.1073/pnas.89.22.10979.

64. Green, S.R., and Mathews, M.B. (1992). Two RNA-binding motifs in the double-stranded RNA-activated protein kinase, DAI. Genes Dev. *6*, 2478–2490. https://doi.org/10.1101/gad.6.12b.2478.

65. Fierro-Monti, I., and Mathews, M.B. (2000). Proteins binding to duplexed RNA: one motif, multiple functions. Trends Biochem. Sci. *25*, 241–246. https://doi.org/10.1016/s0968-0004(00)01580-2.

66. Fu, Q., and Yuan, Y.A. (2013). Structural insights into RISC assembly facilitated by dsRNA-binding domains of human RNA helicase A (DHX9). Nucleic Acids Res. *41*, 3457–3470. https://doi.org/10.1093/nar/gkt042.

67. Gan, J., Tropea, J.E., Austin, B.P., Court, D.L., Waugh, D.S., and Ji, X. (2006). Structural insight into the mechanism of double-stranded RNA processing by ribonuclease III. Cell *124*, 355–366. https://doi.org/10.1016/j.cell.2005.11.034.

68. Masliah, G., Maris, C., Konig, S.L., Yulikov, M., Aeschimann, F., Malinowska, A.L., Mabille, J., Weiler, J., Holla, A., Hunziker, J., et al. (2018). Structural basis of siRNA recognition by TRBP double-stranded RNA binding domains. EMBO J. *37*, e97089. https://doi.org/10.15252/embj.201797089.

69. Amunts, A., Brown, A., Bai, X.C., Llácer, J.L., Hussain, T., Emsley, P., Long, F., Murshudov, G., Scheres, S.H.W., and Ramakrishnan, V. (2014). Structure of the yeast mitochondrial large ribosomal subunit. Science *343*, 1485–1489. https://doi.org/10.1126/science.1249410.

70. Bass, B.L., and Weintraub, H. (1988). An unwinding activity that covalently modifies its double-stranded RNA substrate. Cell *55*, 1089–1098. https://doi.org/10.1016/0092-8674(88)90253-x.

71. Masliah, G., Barraud, P., and Allain, F.H.T. (2013). RNA recognition by double-stranded RNA binding domains: a matter of shape and sequence. Cell. Mol. Life Sci. *70*, 1875–1895. https://doi.org/10.1007/s00018-012-1119-x.

72. Stefl, R., Oberstrass, F.C., Hood, J.L., Jourdan, M., Zimmermann, M., Skrisovska, L., Maris, C., Peng, L., Hofr, C., Emeson, R.B., and Allain, F.H.T. (2010). The solution structure of the ADAR2 dsRBM-RNA complex reveals a sequence-specific readout of the minor groove. Cell *143*, 225–237. https://doi.org/10.1016/j.cell.2010.09.026.

73. Stefl, R., Xu, M., Skrisovska, L., Emeson, R.B., and Allain, F.H.T. (2006). Structure and specific RNA binding of ADAR2 double-stranded RNA binding motifs. Structure *14*, 345–355. https://doi.org/10.1016/j.str.2005.11.013.

74. Thuy-Boun, A.S., Thomas, J.M., Grajo, H.L., Palumbo, C.M., Park, S., Nguyen, L.T., Fisher, A.J., and Beal, P.A. (2020). Asymmetric dimerization of adenosine deaminase acting on RNA facilitates substrate recognition. Nucleic Acids Res. *48*, 7958–7972. https://doi.org/10.1093/nar/gkaa532.

75. Doherty, E.E., Karki, A., Wilcox, X.E., Mendoza, H.G., Manjunath, A., Matos, V.J., Fisher, A.J., and Beal, P.A. (2022). ADAR activation by inducing a syn conformation at guanosine adjacent to an editing site. Nucleic Acids Res. *50*, 10857–10868. https://doi.org/10.1093/nar/gkac897.

76. Barraud, P., Banerjee, S., Mohamed, W.I., Jantsch, M.F., and Allain, F.H.T. (2014). A bimodular nuclear localization signal assembled via an extended double-stranded RNA-binding domain acts as an RNA-sensing signal for transportin 1. Proc. Natl. Acad. Sci. USA *111*, E1852–E1861. https://doi.org/10.1073/pnas.1323698111.

77. Ryter, J.M., and Schultz, S.C. (1998). Molecular basis of double-stranded RNA-protein interactions: structure of a dsRNA-binding domain complexed with dsRNA. EMBO J. *17*, 7505–7513. https://doi.org/10.1093/emboj/17.24.7505.

78. Hol, W.G., van Duijnen, P.T., and Berendsen, H.J. (1978). The alpha-helix dipole and the properties of proteins. Nature *273*, 443–446. https://doi.org/10.1038/273443a0.

79. Menon, S.K., and Lawrence, C.M. (2013). Helix-Turn-Helix Motif. In Brenner's Encyclopedia of Genetics, S. Maloy and K. Hughes, eds. (Academic Press), pp. 412–415. https://doi.org/10.1016/b978-0-12-374984-0.00689-6.

80. Raghava Kurup, R., Oakes, E.K., Manning, A.C., Mukherjee, P., Vadlamani, P., and Hundley, H.A. (2022). RNA binding by ADAR3 inhibits adenosine-to-inosine editing and promotes expression of immune response protein MAVS. J. Biol. Chem. *298*, 102267. https://doi.org/10.1016/j.jbc.2022.102267.

81. Macbeth, M.R., Schubert, H.L., Vandemark, A.P., Lingam, A.T., Hill, C.P., and Bass, B.L. (2005). Inositol hexakisphosphate is bound in the ADAR2 core and required for RNA editing. Science *309*, 1534–1539. https://doi.org/10.1126/science.1113150.

82. Bransteitter, R., Prochnow, C., and Chen, X.S. (2009). The current structural and functional understanding of APOBEC deaminases. Cell. Mol. Life Sci. *66*, 3137–3147. https://doi.org/10.1007/s00018-009-0070-y.

83. Johansson, E., Neuhard, J., Willemoës, M., and Larsen, S. (2004). Structural, kinetic, and mutational studies of the zinc ion environment in tetrameric cytidine deaminase. Biochemistry *43*, 6020–6029. https://doi.org/10.1021/bi035893x.

84. Haudenschild, B.L., Maydanovych, O., Véliz, E.A., Macbeth, M.R., Bass, B.L., and Beal, P.A. (2004). A transition state analogue for an RNA-editing reaction. J. Am. Chem. Soc. *126*, 11213–11219. https://doi.org/10.1021/ja0472073.

85. Kuttan, A., and Bass, B.L. (2012). Mechanistic insights into editing-site specificity of ADARs. Proc. Natl. Acad. Sci. USA *109*, E3295–E3304. https://doi.org/10.1073/pnas.1212548109.

86. Phelps, K.J., Tran, K., Eifler, T., Erickson, A.I., Fisher, A.J., and Beal, P.A. (2015). Recognition of duplex RNA by the deaminase domain of the RNA editing enzyme ADAR2. Nucleic Acids Res. *43*, 1123–1132. https://doi.org/10.1093/nar/gku1345.

87. Doherty, E.E., Wilcox, X.E., van Sint Fiet, L., Kemmel, C., Turunen, J.J., Klein, B., Tantillo, D.J., Fisher, A.J., and Beal, P.A. (2021). Rational Design of RNA Editing Guide Strands: Cytidine Analogs at the Orphan Position. J. Am. Chem. Soc. *143*, 6865–6876. https://doi.org/10.1021/jacs.0c13319.

88. Malik, T.N., Doherty, E.E., Gaded, V.M., Hill, T.M., Beal, P.A., and Emeson, R.B. (2021). Regulation of RNA editing by intracellular acidification. Nucleic Acids Res. *49*, 4020–4036. https://doi.org/10.1093/nar/gkab157.

89. Yang, Z., Hutter, D., Sheng, P., Sismour, A.M., and Benner, S.A. (2006). Artificially expanded genetic information system: a new base pair with an alternative hydrogen bonding pattern. Nucleic Acids Res. *34*, 6095–6101. https://doi.org/10.1093/nar/gkl633.

90. Eggington, J.M., Greene, T., and Bass, B.L. (2011). Predicting sites of ADAR editing in double-stranded RNA. Nat. Commun. *2*, 319. https://doi.org/10.1038/ncomms1324.

91. Eifler, T., Pokharel, S., and Beal, P.A. (2013). RNA-Seq analysis identifies a novel set of editing substrates for human ADAR2 present in *Saccharomyces cerevisiae*. Biochemistry *52*, 7857–7869. https://doi.org/10.1021/bi4006539.

92. Schneider, M.F., Wettengel, J., Hoffmann, P.C., and Stafforst, T. (2014). Optimal guideRNAs for re-directing deaminase activity of hADAR1 and hADAR2 in trans. Nucleic Acids Res. *42*, e87. https://doi.org/10.1093/nar/gku272.

93. Wang, Y., Park, S., and Beal, P.A. (2018). Selective Recognition of RNA Substrates by ADAR Deaminase Domains. Biochemistry *57*, 1640–1651. https://doi.org/10.1021/acs.biochem.7b01100.

94. Park, S., Doherty, E.E., Xie, Y., Padyana, A.K., Fang, F., Zhang, Y., Karki, A., Lebrilla, C.B., Siegel, J.B., and Beal, P.A. (2020). High-throughput mutagenesis reveals unique structural features of human ADAR1. Nat. Commun. *11*, 5130. https://doi.org/10.1038/s41467-020-18862-2.

95. Brinkman, H.F., Jauregui Matos, V., Mendoza, H.G., Doherty, E.E., and Beal, P.A. (2023). Nucleoside analogs in ADAR guide strands targeting 5'-UA sites. RSC Chem. Biol. *4*, 74–83. https://doi.org/10.1039/d2cb00165a.

96. Oakes, E., Anderson, A., Cohen-Gadol, A., and Hundley, H.A. (2017). Adenosine Deaminase That Acts on RNA 3 (ADAR3) Binding to Glutamate Receptor Subunit B Pre-mRNA Inhibits RNA Editing in Glioblastoma. J. Biol. Chem. *292*, 4326–4335. https://doi.org/10.1074/jbc.M117.779868.

97. Valente, L., and Nishikura, K. (2007). RNA binding-independent dimerization of adenosine deaminases acting on RNA and dominant negative effects of nonfunctional subunits on dimer functions. J. Biol. Chem. *282*, 16054–16061. https://doi.org/10.1074/jbc.M611392200.

98. Li, M., Yan, C., Jiao, Y., Xu, Y., Bai, C., Miao, R., Jiang, J., and Liu, J. (2022). Site-directed RNA editing by harnessing ADARs: advances and challenges. Funct. Integr. Genomics *22*, 1089–1103. https://doi.org/10.1007/s10142-022-00910-3.

99. Booth, B.J., Nourreddine, S., Katrekar, D., Savva, Y., Bose, D., Long, T.J., Huss, D.J., and Mali, P. (2023). RNA editing: Expanding the potential of RNA therapeutics. Mol. Ther. *31*, 1533–1549. https://doi.org/10.1016/j.ymthe.2023.01.005.

100. Diaz Quiroz, J.F., Siskel, L.D., and Rosenthal, J.J.C. (2023). Site-directed A–> I RNA editing as a therapeutic tool: moving beyond genetic mutations. RNA *29*, 498–505. https://doi.org/10.1261/rna.079518.122.

101. Merkle, T., and Stafforst, T. (2021). New Frontiers for Site-Directed RNA Editing: Harnessing Endogenous ADARs. Methods Mol. Biol. *2181*, 331–349. https://doi.org/10.1007/978-1-0716-0787-9_19.

OMTN, Volume *35*

Supplemental information

Structural perspectives on adenosine to inosine RNA editing by ADARs

Andrew J. Fisher and Peter A. Beal

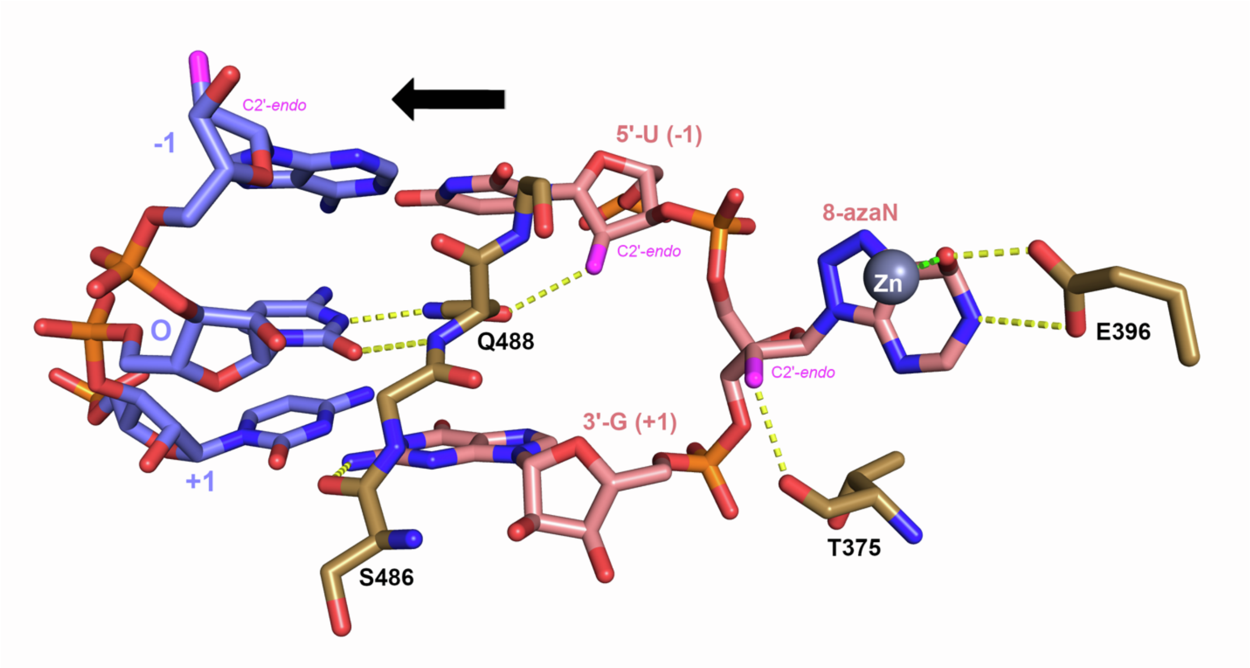

Figure S1. *Detailed RNA-Protein contacts near the flipped-out base. Edited RNA strand is shown in salmon-colored carbon atoms, guide strand RNA in blue-colored carbon. RNA with 2'C-endo sugar puckers are highlighted with magenta-colored 2'-O atom and labeled. Guild strand RNA numbering shown in blue with the -1 position base-pairing to the 5'-U of the edited strand. Bold black arrow shows "sliding" of 5'-end base-pair relative to ideal A-form conformation. Yellow dashed lines show hydrogen bonding, green dash, zinc ligation.*

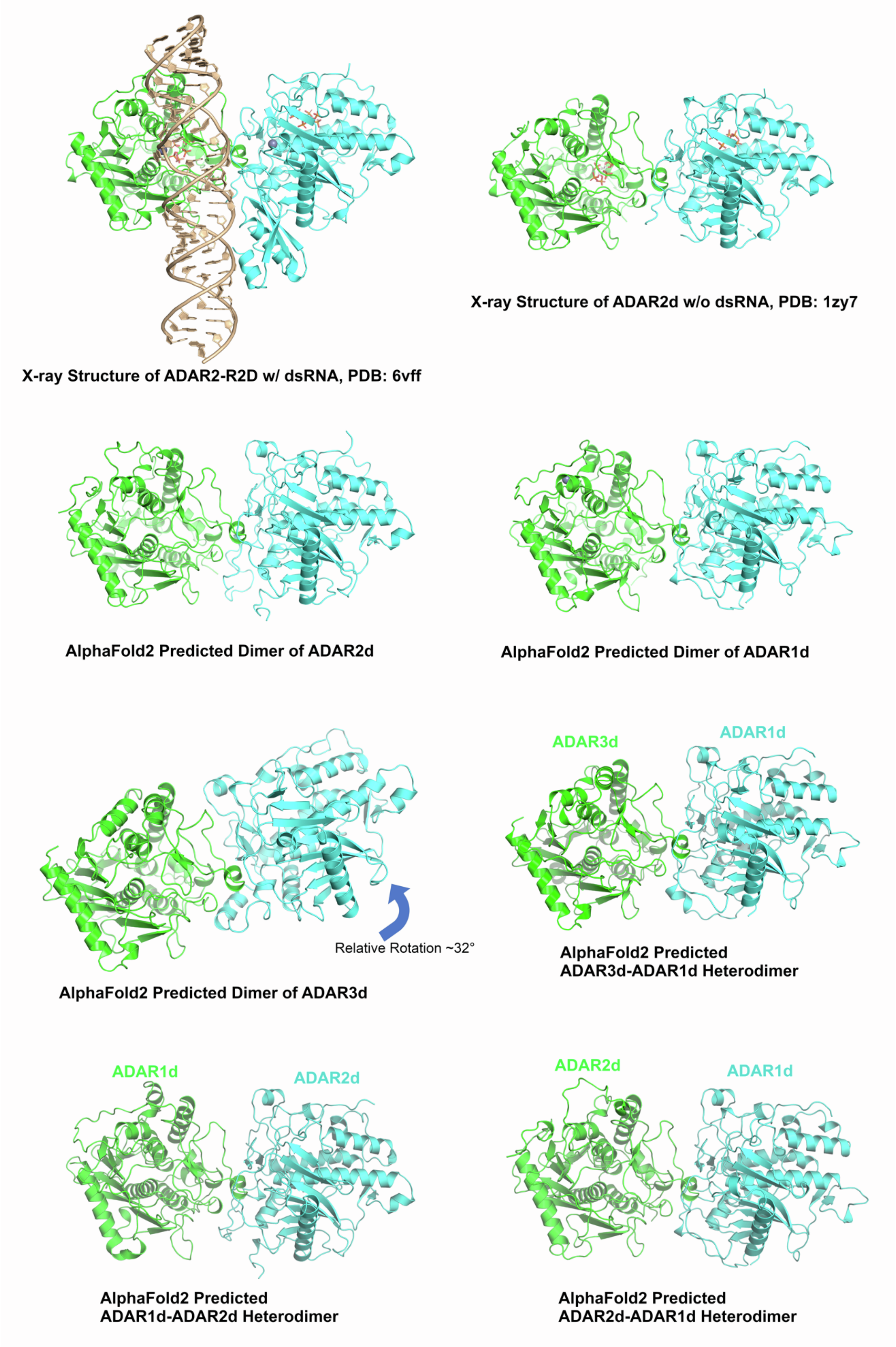
X-ray Structure of ADAR2-R2D w/ dsRNA, PDB: 6vff
X-ray Structure of ADAR2d w/o dsRNA, PDB: 1zy7
AlphaFold2 Predicted Dimer of ADAR2d
AlphaFold2 Predicted Dimer of ADAR1d
Relative Rotation ~32°
AlphaFold2 Predicted Dimer of ADAR3d
ADAR3d
ADAR1d
AlphaFold2 Predicted
ADAR3d-ADAR1d Heterodimer
ADAR1d
ADAR2d
AlphaFold2 Predicted
ADAR1d-ADAR2d Heterodimer
ADAR2d
ADAR1d
AlphaFold2 Predicted
ADAR2d-ADAR1d Heterodimer

Figure S2. *Dimeric arrangement of ADARs. Shown are different ADAR structures determined experientially and modeled by AlphaFold2 showing the dimerization helix mediates similar asymmetric homodimers from different ADARs. Green-colored monomers reside in the catalytic position whose active site would bind the flipped-out adenosine, and the cyan-colored monomers represent the auxiliary position, whose active site binds the catalytic monomer's dimerization helix. AlphaFold2 also predicts an ADAR3d-ADAR2d heterodimer, but this not shown.*

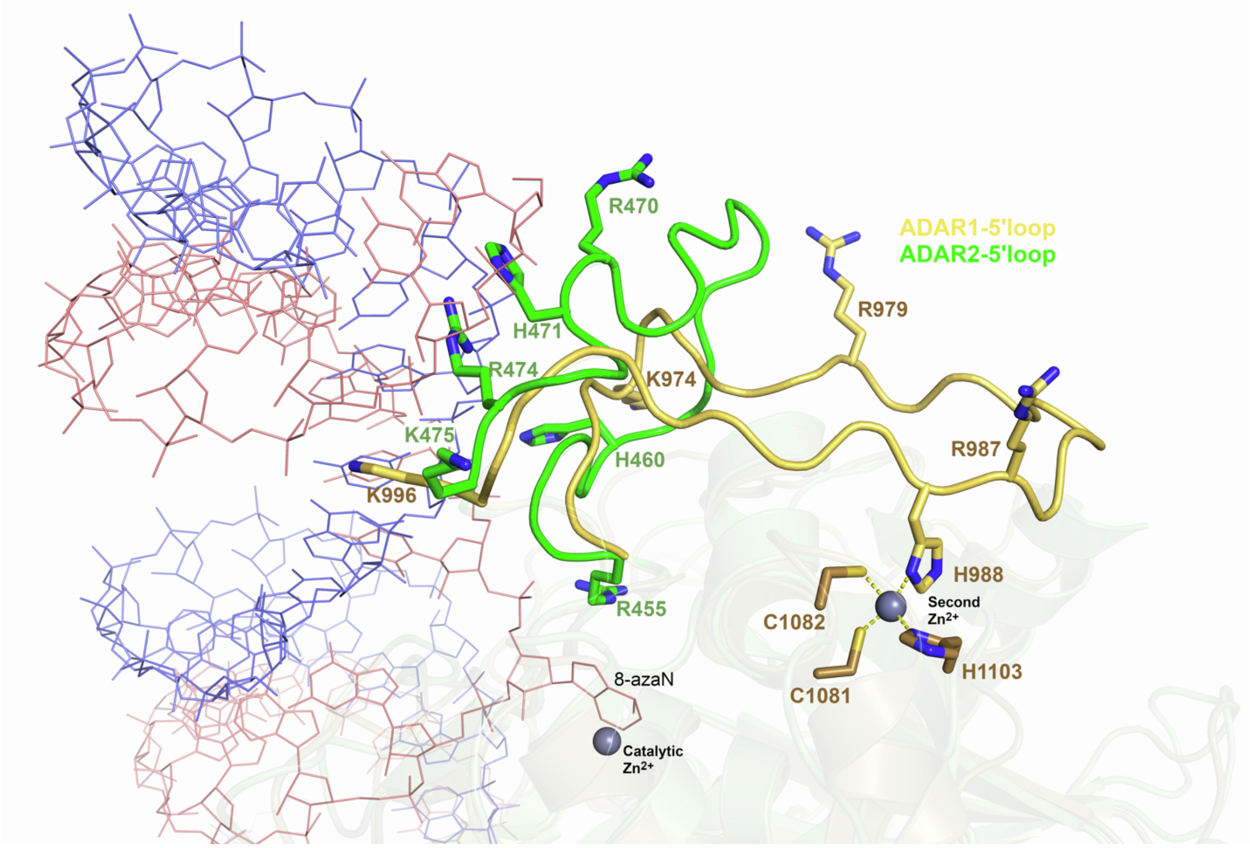

Figure S3. *Structures of the 5'-RNA binding loop as seen in the crystal structure of ADAR2 (Green, PDBID: 6vff), and the AlphaFold2 predicted structure of human ADAR1 (yellow). Only the loops are highlighted with the rest of the protein structures shown in transparent cartoon (green and brown for ADAR2 and ADAR1, respectively) and the RNA in the ADAR2-RD structure displayed in thin lines. Conserved residues in the 5'-RNA binding loop for ADAR2 that interact with RNA are shown as stick sidechains. Positively-charged conserved residues in ADAR1 are also shown with stick sidechains.*
